# A chromatography-free and aqueous waste-free process for thioamide preparation with Lawesson’s reagent

**DOI:** 10.3762/bjoc.17.69

**Published:** 2021-04-09

**Authors:** Ke Wu, Yichen Ling, An Ding, Liqun Jin, Nan Sun, Baoxiang Hu, Zhenlu Shen, Xinquan Hu

**Affiliations:** 1 College of Chemical Engineering, Zhejiang University of Technology, Hangzhou 310032, P.R. China

**Keywords:** chromatography-free, Lawesson’s reagent, scale-up, thioamide, thionation

## Abstract

After completing the thio-substitution with Lawesson’s reagent, ethanol was found to be effective in the decomposition of the inherent stoichiometric six-membered-ring byproduct from the Lawesson’s reagent to a highly polarized diethyl thiophosphonate. The treatment significantly simplified the following chromatography purification of the desired thioamide in a small scale preparation. As scaling up the preparation of two pincer-type thioamides, we have successfully developed a convenient process with ethylene glycol to replace ethanol during the workup, including a traditional phase separation, extraction, and recrystallization. The newly developed chromatography-free procedure did not generate P-containing aqueous waste, and only organic effluents were discharged. It is believed that the optimized procedure offers the great opportunity of applying the Lawesson’s reagent for various thio-substitution reactions on a large scale.

## Introduction

**Figure 1:**
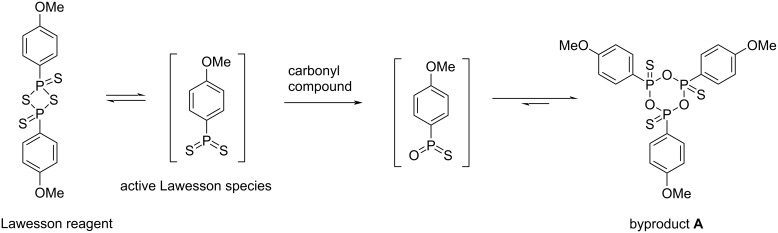
Generation of the six-membered byproduct **A** from thionation reactions using Lawesson’s reagent.

The transformation of a carbonyl into a thiocarbonyl group is one of the most important reactions in organic synthetic chemistry [1]. Lawesson’s reagent (LR) is widely applied in this transformation, as well as for the syntheses of various sulfur-containing heterocyclic compounds [2–5]. Although LR is a powerful, mild and versatile thionation reagent, the workup procedures of reactions involving this reagent have received quite a few negative comments [6–10]. The reason for this is that an inherent six-membered ring structure **A** (Figure 1) is formed from the LR upon thio-substitution [11–13]. It has been observed that the polarity of compound **A** is generally similar to the desired products, thus making the purification of the desired products by extraction operations less efficient. Therefore, the purification is rather difficult and usually a separation by careful column chromatography is necessary because of both the similar polarity and the stoichiometric amount of the formed compound **A**. With regard to this, the use of LR was always limited to small-scale preparations [6,8].

**Figure 2:**
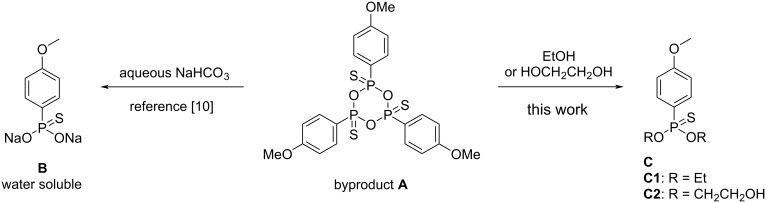
Work-up procedure for the reaction with LR.

Other than the exploration of potential surrogates of LR for the thionation reactions, considerable efforts have been devoted to the improvement of the workup procedures of these reactions. For example, Soós and co-workers introduced perfluoroalkylated derivatives of LR, which simplified the product isolation via a fluorous reversed-phase solid extraction technique [9–10]. This method with long perfluorinated alkyl chains is attractive in parallel synthesis and also in high-throughput biological screening. However, both the modified LR and the fluorous solvents are rather expensive and not practical for scaling up. Besides, basic aqueous solutions were utilized as well in the work-up process and it was believed that the compound **A** was converted to a water-soluble thiophosphonate (Figure 2) [14–16]. Although this operation simplified the work-up procedure and allowed a scaled up chromatography-free purification, it generated quite large amounts of P-containing aqueous waste. The P-containing aqueous waste is unfavorable during the scaling up because of the difficult treatment in the downstream and also because it is one of the sources of eutrophication. Therefore, optimizing the work-up process of LR-mediated thionation reactions is greatly appealing for potential large-scale preparations.

Thioamides are highly attractive molecules in pharmaceuticals, agrochemicals, electronic chemicals, and materials sciences [17–27]. In coordination chemistry, pincer-type ligands containing a thioamide motif were shown to exhibit incomparable chelating ability towards selected transition metals, and the corresponding complexes were applied in various areas such as chemical-sensor materials, tunable redox-potential complexes, polymer hybrid luminescence materials, building blocks for multinuclear complexes, and as catalysts for cross-coupling reactions [28–35]. The thio-substitution of amides with LR is an efficient and straightforward method, because the amide substrates and LR are readily available and the reactions are easily operated [8–9,36–40]. With thio-substitution of amides as a model, we herein reported an efficient work-up procedure of applying LR by utilizing ethylene glycol to decompose the compound **A** (Figure 2). With a combination of some usual operations, such as, phase separation, extraction and crystallization, the desired thioamide products were efficiently obtained in excellent yields.

## Results and Discussion

Our initial exploration began with the thio-substitution of *N*-phenylbenzamide (**1a**) with LR in toluene at reflux (Table 1). After completing the reaction, the solutions were split and treated with different additives at various temperatures in order to identify a reagent that efficiently decomposes compound **A** [41]. MeOH and EtOH were initially tested. The experimental results showed that MeOH can’t fully decompose compound **A** in a temperature range between 40 °C to reflux, either after the removal of toluene or not. To our delight, EtOH worked well at reflux temperature and a new spot with a much higher polarity was observed on the TLC plate. With a mixed solvent of ethyl acetate/petroleum ether 1:3 as the eluent, the *R*_f_ of compound **A** was around 0.5, while the *R*_f_ of the newly generated compound was at around 0.05 either from MeOH or EtOH treatment. Later, the new compound obtained by EtOH treatment was assigned as diethyl (*p*-methoxyphenyl)thiophosphonate (**C1**) via GC–MS and confirmed by GC–TOF [42]. The change in the polarity of the byproduct after MeOH or EtOH treatment indicated that the separation by column chromatography could be simplified in a small-scale preparation. Indeed, with this improvement, various thioamides were conveniently synthesized and isolated in good to excellent yields after column chromatography (Table 1).

**Table 1:** Synthesis of thioamides ^a^.

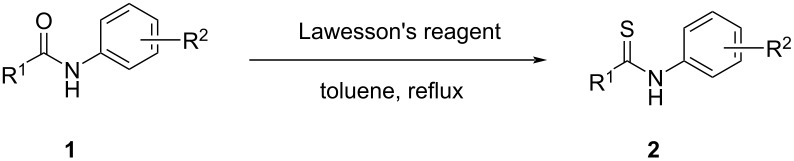					

entry	substrate			product **2**	yield (%)^b^

	R^1^	R^2^	**1**		

1	Ph	H	**1a**	**2a**	85
2	4-MeC_6_H_4_	H	**1b**	**2b**	79
3	4-BrC_6_H_4_	H	**1c**	**2c**	79
4	4-*t-*BuC_6_H_4_	H	**1d**	**2d**	76
5	Ph	4-Me	**1e**	**2e**	82
6	Ph	4-Cl	**1f**	**2f**	92
7	Ph	4-Br	**1g**	**2g**	84
8	Ph	4-I	**1h**	**2h**	78
9	Ph	3-Cl	**1i**	**2i**	75
10	PhCH_2_	H	**1j**	**2j**	70
11	*t*-Bu	H	**1k**	**2k**	76

^a^Reaction conditions: **1** (1 mmol), LR (0.55 mmol), toluene (4 mL), reflux, 4 h, then 2 mL EtOH, reflux, 2 h; ^b^isolated yields after column chromatography.

Although the synthesis of the thioamides at a 1 mmol scale was successful, the required purification by column chromatography is a major drawback for reactions at a larger scale. All attempts to isolate the desired product from the reaction mixture after ethanol treatment, e.g., by solvent extraction/phase separation using solvents of various polarity or even with an aqueous workup failed and showed that compound **C1** was well distributed in most solvents and acted as a polar solvent component. Although less polar solvents, such as heptane, reduced the amount of compound **C1**, however, they also failed to dissolve the desired thioamide product. Water as the solvent could partially extract compound **C1**, but the aqueous phase contained some product, too. Moreover, an aqueous workup after the EtOH treatment is regarded problematic because of the produced P-containing aqueous waste and this two-step treatment procedure was not as efficient as the direct workup with a saturated aqueous NaHCO_3_ solution [15]. With the above observations, we reckoned that converting compound **A** to a more polar derivative could probably be a breakthrough. As common knowledge, the more polarized alcohols over EtOH are those diols or polyols, while MeOH was previously ruled out. Ethylene glycol, a basic chemical and the simplest diol, is slightly soluble in toluene and attracted our interest. Both, its strong polarity and layering ability suggested that ethylene glycol may be an ideal choice.

**Figure 3:**
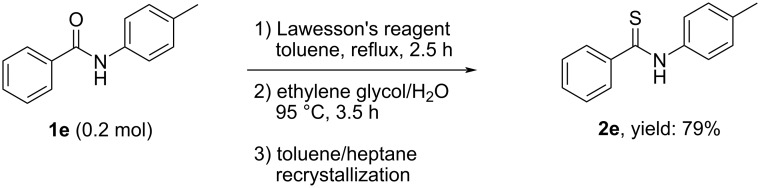
Modified process for the synthesis of **2e** via phase separation.

Therefore, after completion of the thio-substitution reaction of **1e** (0.20 mol) with LR (0.102 mol, Figure 3), excess ethylene glycol was added to decompose compound **A** following the same procedure as described above for the EtOH treatment. To our surprise, the decomposition was much slower as expected (TLC monitoring, see Supporting Information File 1). It was assumed that the ring-opening could be influenced by water or by the in situ-generated thiophosphonic acid. Thus, in addition to ethylene glycol 1.0 mL of water was added to the mixture and we were pleased to find that compound **A** smoothly decomposed at 95 °C in 3.5 h. With the decline of compound **A** in the toluene layer, a new compound **C2** emerged. It was also noticed that the pH value of the ethylene glycol layer was about 2–3. Thus, we reckoned that the assumed byproduct **C2** was further decomposed to the thiophosphonic acid which is well soluble in ethylene glycol. After phase separation at ≈50 °C, the ethylene glycol phase was back-extracted with toluene. The cooled toluene layers were treated with activated carbon and filtered. Then, toluene and other potential volatiles were removed, the residue crystallized from a toluene and heptane solvent mixture to afford 36.0 g (97%) of the desired thioamide **2e** as yellow crystalline solid.

**Scheme 1:**
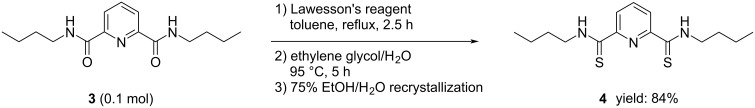
Modified process for the synthesis of pincer ligand **4**.

**Scheme 2:**
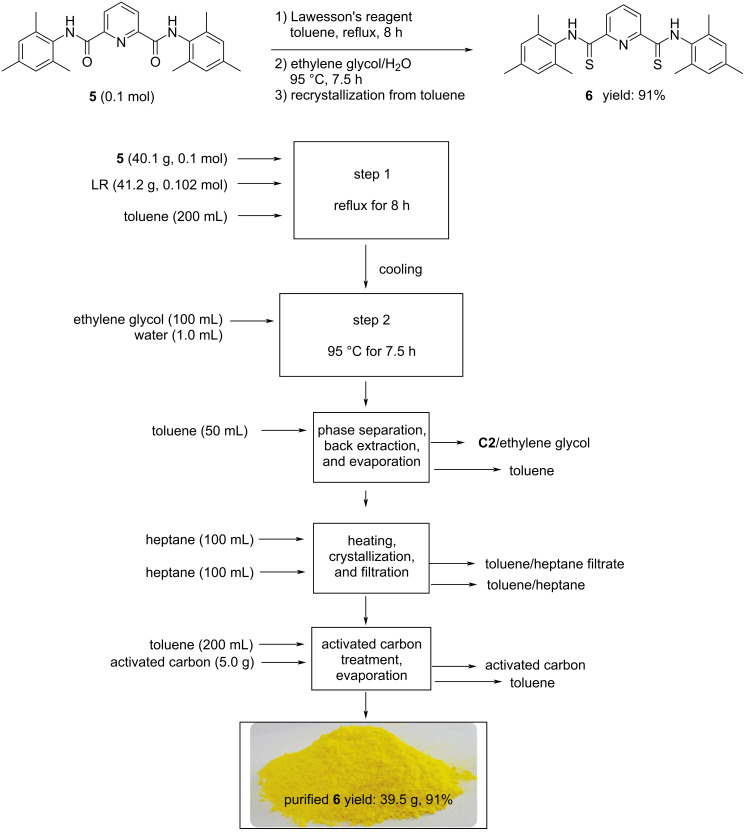
Modified process for the synthesis of pincer-type ligand **6**.

With the proof that ethylene glycol can efficiently decompose compound **A** and simplify the work-up of the reaction with LR, this newly developed method was extended to synthesize two pincer ligands, *N*^2^,*N*^6^-di(*n*-butyl)pyridine-2,6-bis(carbothioamide) (**4**, Scheme 1) and *N*^2^,*N*^6^-bis(2,4,6-trimethylphenyl)pyridine-2,6-bis(carbothioamide) (**6**, Scheme 2) [34–35,43–44]. With a slight excess of LR, the diamide substrate **3** underwent thio-substitution in refluxing toluene for 2.5 h, as monitored by TLC. After cooling of the reaction mixture, 100 mL of ethylene glycol containing 1.0 mL of water were added and the mixture stirred at 95 °C (oil bath) for 5 h. Then, the mixture was allowed to cool to about 50 °C and transferred into a separation funnel for phase separation. The lower ethylene glycol phase was separated and extracted with additional 50 mL of toluene. The combined toluene layers were then treated with 3.1 g of activated carbon (10 wt % of theoretical product) at room temperature. After filtration, the yellow-colored toluene solution was concentrated and the residue recrystallized from 75% EtOH/water to afford 26.1 g (84%) of the desired product **4** as yellow crystalline solid.

Because of a relative lower solubility and the higher molecular weight of diamide substrate **5**, a longer time for the reaction with LR was essential for the completion of the reaction according to TLC monitoring. Following the similar workup procedure as described for compound **4**, the resulting crude product was recrystallized from toluene to afford the product as bright-yellow crystalline solid in 91% yield. The overall preparation of the pincer-type compound **6** is shown in Scheme 2.

As can be seen from Scheme 2, it is clear that the modified process only discharged organic wastes (toluene, and toluene/heptane mixture), **C2**/ethylene glycol mixture, along with some waste of activated carbon [45].

## Conclusion

In conclusion, we have developed a highly efficient process for the workup of thio-substitution reactions with Lawesson’s reagent. In the newly developed procedure, ethylene glycol played a crucial role in the chromatography-free and avoiding P-containing aqueous waste workup procedure. With the preparation of a range of thioamides as examples, the ethylene glycol treatment allowed the work-up process involving phase separation, back-extraction, activated carbon treatment, and final recrystallization from a proper solvent. Parts of the recovered solvent could be reused and the effluent was also reduced. The improved procedure is believed to be suitable for the large-scale preparations with the application of Lawesson’s reagent.

## Experimental

### General

The NMR spectra were recorded on a 400 MHz, 500 MHz, and 600 MHz spectrometer in deuterated solvents using tetramethylsilane (TMS) as an internal standard. Chemical shifts were reported in parts per million (ppm, δ) downfield from TMS. The following abbreviations were used to explain the multiplicities: s = singlet, d = doublet, t = triplet, q = quartet, m = multiplet, br = broad. The structures of compounds **2a–l** were confirmed by comparison with reference data. Melting points were determined on a Büchi M-565 apparatus. All reagents were obtained from commercial suppliers and used without further purification. The reactions were monitored by thin-layer chromatography. Column chromatography was performed using silica gel (300–400 mesh). Amides **1a**–**l** were prepared following the reported procedure [46].

#### Typical procedure for the synthesis of thioamide **2** (1 mmol scale)

A mixture of amide **1** (1.0 mmol) and Lawesson’s reagent (0.60 mmol) was refluxed in toluene (4 mL). The reaction progress was monitored by TLC until full consumption of the starting amide was observed. To the cooled mixture was added EtOH (2 mL, excess) and the resulting mixture was heated at reflux for 2 h. Then, the volatiles were removed under reduced pressure. The residue was diluted with ethyl acetate followed by aqueous workup. The organic phase was dried over anhydrous MgSO_4_. The solvent was removed under reduced pressure. The reside was purified by silica gel column chromatography using petroleum ether/ethyl acetate as the eluent to afford the desired thioamide **2**.

#### Synthesis of *N*^2^,*N*^6^-di(*n*-butyl)pyridine-2,6-(carbothioamide) (**3**) [34]

To a 500 mL four-necked flask were added 50.1 g of pyridine-2,6-dicarboxylic acid (0.3 mol), 1 mL of DMF and 55 mL of SOCl_2_. The mixture was heated to 80 °C to get a clear solution and stirred for 30 min. Then, SOCl_2_ was removed in vacuum and the crude acyl chloride was dissolved in 50 mL of toluene. To a 1 L flask were added 48 g of *n*-butylamine (0.66 mol), 126 g of NaHCO_3_ (1.5 mol), 400 mL of toluene and 100 mL of water. At 0 °C, the above acyl chloride in toluene was added slowly and the mixture was stirred for 20 min at 0 °C and allowed to reach room temperature over 30 min. After filtration, the filter cake was washed with water and recrystallized from EtOH (200 mL)/H_2_O (90 mL) to afford the desired diamide **3** in 70% yield as white solid. Mp 155.2–158.0 °C; ^1^H NMR (600 MHz, CDCl_3_) δ 8.33 (d, *J* = 7.8 Hz, 2H), 8.03–7.94 (m, 3H), 3.48–3.42 (m, 4H), 1.63–1.55 (m, 4H), 1.42–1.32 (m, 4H), 0.93–0.89 (m, 6H); ^13^C NMR (150 MHz, CDCl_3_) δ 163.7, 149.1, 139.1, 125.0, 39.5, 31.9, 20.3, 13.9.

#### Synthesis of *N*^2^,*N*^6^-bis(2,4,6-trimethylphenyl)pyridine-2,6-(carbothioamide) (**5**) [34]

Following a similar procedure as described for diamide **3**, the desired diamide **5** was obtained from the reaction of pyridine-2,6-dicarboxylic acid (50.1 g, 0.3 mol) and 2,4,6-trimethylaniline (89.2 g, 0.66 mol) in 92% yield as white solid. Mp 191.5–193.3 °C; ^1^H NMR (600 MHz, CDCl_3_) δ 9.05 (s, 1H), 8.51 (d, *J* = 7.8 Hz, 2H), 8.15 (t, *J* = 7.8 Hz, 1H), 6.96 (s, 4H), 2.31 (s, 6H), 2.26 (s, 12H); ^13^C NMR (150 MHz, CDCl_3_) δ 161.8, 149.0, 139.5, 137.3, 135.1, 130.7, 129.2, 125.7, 77.4, 77.2, 77.0, 21.1, 18.5.

#### Procedure for the synthesis of *N*-(*p*-methylphenyl)benzothioamide (**2e**) (0.2 mol scale) [47]

To a 500 mL three-necked flask, 42.3 g of *N*-*p*-methylphenylbenzamide (0.20 mol), 42.0 g of Lawesson’s reagent (0.104 mol), and 200 mL of toluene were added. The mixture was heated to reflux under a nitrogen atmosphere. The reaction was completed in 3 h by TLC monitoring. Then, to the cooled mixture, were added 100 mL of ethylene glycol (excess), together with 1.0 mL of water, and the resulting mixture was stirred at 95 °C. TLC monitoring of the toluene layer showed that the byproduct **A** from Lawesson’s reagent had disappeared after 3.5 h. The slightly cooled mixture was transferred to a separation funnel. The ethylene glycol layer was left standing overnight. The formed precipitate was collected, combined with the toluene phase and heated to form a clear solution. Then, 50 mL of heptane were added and the resulting solution was gradually cooled. The precipitation was observed at 65 °C and stirring was continued until the temperature reached 20 °C. After filtration, the solid was washed with heptane (50 mL) and dried to afford 36.0 g of the desired thioamide product **2e** (79%) as bright yellow crystalline solid. Mp 128.5–130.1 °C; ^1^H NMR (500 MHz, DMSO-*d_6_*) δ 11.67 (s, 1H), 7.82 (d, *J* = 7.6 Hz, 2H), 7.69 (d, *J* = 7.6 Hz, 2H), 7.52 (t, *J* = 7.0 Hz, 1H), 7.46 (t, *J* = 7.6 Hz, 2H), 7.24 (d, *J* = 7.6 Hz, 2H), 2.32 (s, 3H); ^13^C NMR (125 MHz, DMSO-*d_6_*) δ 197.4, 142.7, 137.6, 135.7, 130.7, 129.0, 128.1, 127.5, 124.2, 20.8.

#### Procedure for the synthesis of *N*^2^,*N*^6^-di(*n*-butyl)pyridine-2,6-bis(carbothioamide) (**4**) (0.1 mol scale) [34]

To a 500 mL three-necked flask, 27.7 g of *N*^2^,*N*^6^-di(*n*-butyl)pyridine-2,6-(carbothioamide) (**3**, 0.10 mol), 42.4 g Lawesson’s reagent (0.0525 mol), and 150 mL of toluene were added. The mixture was heated to reflux under a nitrogen atmosphere. The reaction was completed in 2.5 h by TLC monitoring. Then, to the cooled mixture were added 100 mL of ethylene glycol (excess), together with 1.0 mL of water, and the resulting mixture was stirred at 95 °C. TLC monitoring of the toluene layer showed that the byproduct **A** from Lawesson’s reagent had disappeared after 5 h. The slightly cooled mixture was transferred to a separation funnel. The ethylene glycol layer and 50 mL of toluene were transferred back to the flask and stirred at 95 °C (oil bath) for 30 min. The combined toluene layers were treated with 3.1 g of activated carbon (10 wt % of the theoretical amount of dithioamide **4**). After filtration, the solid was washed with toluene. The solvent was removed under reduced pressure and the yellowish residue was recrystallized from 190 mL of 75% EtOH to afford 26.1 g of the dithioamide **4** (84%) as a yellowish crystalline solid. Mp 72.2–74.5 °C; ^1^H NMR (600 MHz, CDCl_3_) δ 9.44 (s, 2H), 8.78 (d, *J* = 7.8 Hz, 2H), 7.94 (t, *J* = 7.8 Hz, 1H), 3.91–3.86 (m, 4H), 1.84–1.77 (m, 4H), 1.55–1.48 (m, 4H), 1.02 (t, *J* = 7.4 Hz, 6H); ^13^C NMR (150 MHz, CDCl_3_) δ 190.4, 149.6, 138.4, 127.3, 45.9, 30.2, 20.5, 13.9.

#### Procedure for the synthesis of *N*^2^,*N*^6^-bis(2,4,6-trimethylphenyl)pyridine-2,6-bis(carbothioamide) (**6**) (0.1 mol scale) [34]

To a 500 mL three-necked flask 40.1 g of *N*^2^,*N*^6^-bis(2,4,6-trimethylphenyl)pyridine-2,6-(carbothioamide) (**5**, 0.10 mol), 41.2 g of Lawesson’s reagent (0.051 mol), and 200 mL of toluene were added. The mixture was heated to reflux under a nitrogen atmosphere. TLC monitoring showed that there still existed some amide-thioamide intermediate after 5 h and heating was continued for another 3 h. Afterwards, TLC monitoring showed that the amide-thioamide had nearly disappeared. Then, to the cooled mixture, were added 100 mL of ethylene glycol (excess), together with 1.0 mL of water and the resulting mixture was stirred at 95 °C. TLC monitoring of the toluene layer showed that the byproduct **A** of the Lawesson’s reagent had disappeared after 7.5 h. The slightly cooled mixture was transferred to a separation funnel. The toluene layer was separated, and the ethylene glycol layer and 50 mL of toluene were transferred back to the flask and stirred at 95 °C (oil bath) for 30 min. The toluene layers were combined, part of the solvent removed under reduced pressure and the remaining solution was heated to reflux and diluted with 100 mL of heptane. The solution remained clear during reflux. Then, the solution was cooled to precipitate the crystalline solid that was collected by filtration and washed with heptane (100 mL). Then, the solid was treated with 200 mL of toluene and 5.0 g of activated carbon at 80 °C, followed by hot filtration. The filtrate was concentrated to afford 39.5 g of the desired dithioamide **6** (91%) as bright-yellow crystalline solid. Mp 190.5–193.6 °C; ^1^H NMR (600 MHz, CDCl_3_) δ 10.71 (s, 2H), 8.99 (d, *J* = 7.8 Hz, 2H), 8.07 (t, J = 7.8 Hz, 1H), 6.99 (s, 4H), 2.33 (s, 6H), 2.23 (s, 12H);^13^C NMR (150 MHz, CDCl_3_) δ 190.8, 149.4, 138.7, 138.5, 135.1, 133.4, 129.4, 128.0, 21.3, 18.3.

## Supporting Information

File 1 NMR data for compounds **2**–**6** and thiophosphonic acid.
