# Supplementary material for: A chromatography-free and aqueous waste-free process for thioamide preparation with Lawesson’s reagent
Source: Beilstein J Org Chem. 2021 Apr 9;17:805–12. doi: 10.3762/bjoc.17.69 (PMC8042485; doi:10.3762/bjoc.17.69)
Supplement: File 1 — NMR data for compounds 2–6 and thiophosphonic acid. [file Beilstein_J_Org_Chem-17-805-s001.pdf]

BEILSTEIN JOURNAL OF ORGANIC CHEMISTRY

Supporting Information

for

A chromatography-free and aqueous waste-free process for thioamide preparation with Lawesson's reagent

Ke Wu, Yichen Ling, An Ding, Liqun Jin, Nan Sun, Baoxiang Hu, Zhenlu Shen and Xinquan Hu

Beilstein J. Org. Chem. **2021,** *17,* 805–812. doi:10.3762/bjoc.17.69

NMR data for compounds 2–6 and thiophosphonic acid

License and Terms: This is a supporting information file under the terms of the Creative Commons Attribution License (https://creativecommons.org/licenses/by/4.0). Please note that the reuse, redistribution and reproduction in particular requires that the author(s) and source are credited and that individual graphics may be subject to special legal provisions.
The license is subject to the *Beilstein Journal of Organic Chemistry* terms and conditions: (https://www.beilstein-journals.org/bjoc/terms)

Table of contents

1. Modified process for the synthesis of **2e**.
2. NMR data of amides **2–6** and thiophosphonic acid
3. References

1. Modified process for the synthesis of 2e.

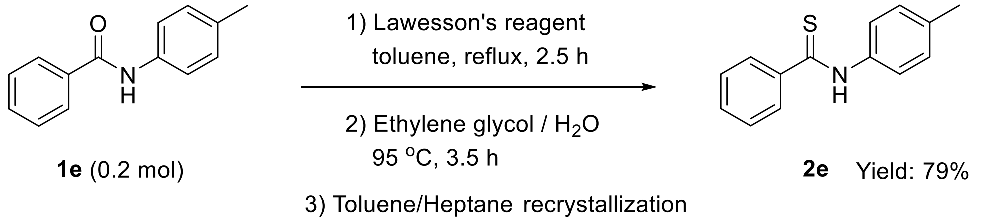

(2)

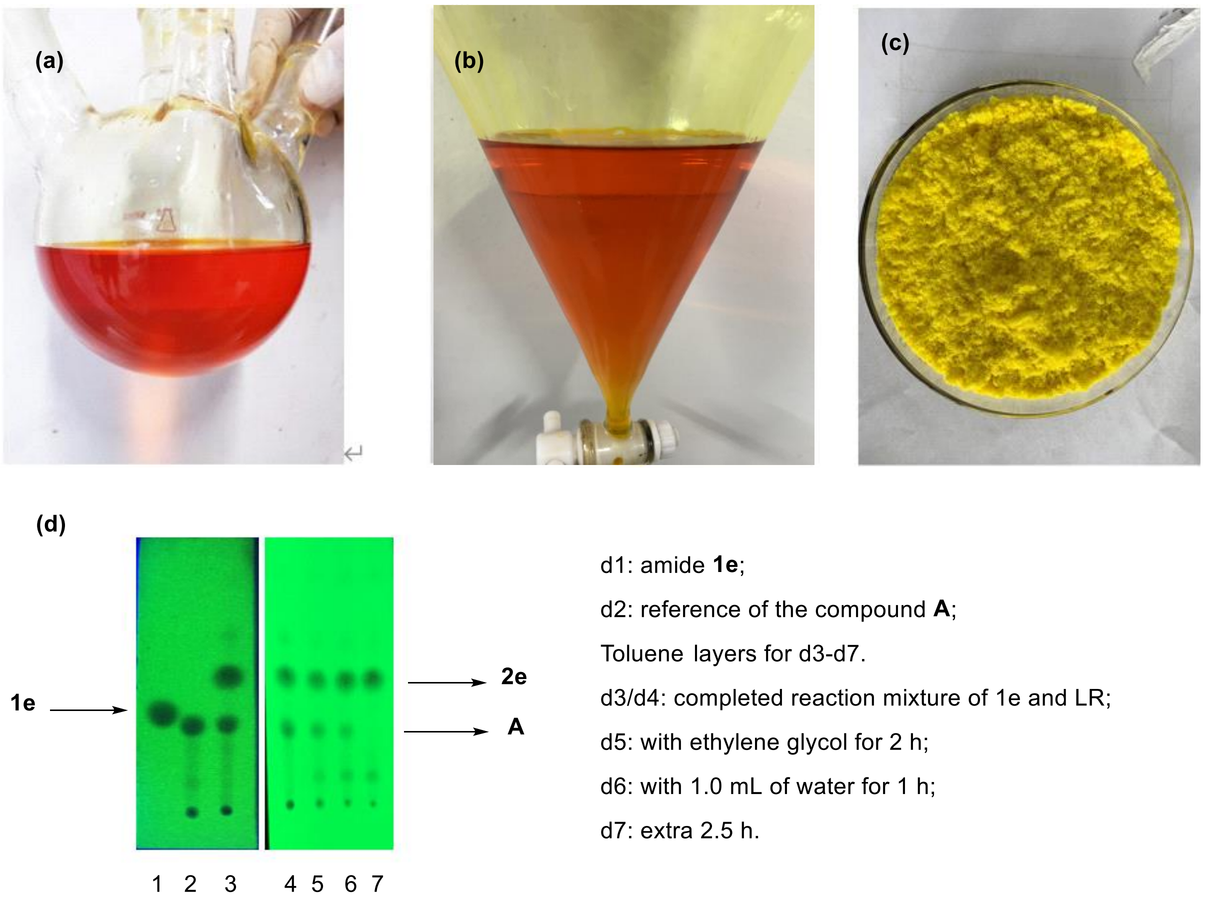

Figure S1: Phase-separation of the reaction mixture, recrystallization of the product, and TLC monitoring of the treatment with ethylene glycol. (a: Complete reaction mixture (upper layer is toluene); b: back extraction (upper layer is toluene); c: product **2e** after recrystallization; d: TLC monitoring of the reaction mixture and reaction workup between amide **1e** and LR).

After the completion of the thioamidation reaction of **1e** (0.20 mol) and LR (0.102 mol) (Figure S1, d3 and d4), excess ethylene glycol was added to decompose compound **A**, following the previous EtOH treatment procedure. To our astonishment, the decomposition was much slower as expected by TLC monitoring (Figure S1, d5). It was assumed that the ring-opening could be interfered by water or accelerated by the in-situ generated thiophosphonic acid. Thus, 1.0 mL of water was added into the mixture. We were pleased to find that the compound **A** smoothly decomposed by water-containing ethylene glycol at 95 °C

within 3.5 h. With the decline of compound **A** in the toluene layer, a new compound **C2** was observed (Figure S1, d7). It was also noticed that the pH of the ethylene glycol layer was about 2–3. Thus, we reckoned that the assumed byproduct **C2** was further decomposed to the thiophosphonic acid which is well soluble in ethylene glycol. After phase separation at around 50 °C (Figure S1, a), the ethylene glycol phase was back-extracted with toluene (Figure S1, b). The cooled toluene layers were treated with activated carbon and filtered. Toluene and the potential volatiles were removed, the residue recrystallized from a mixture of toluene and heptane to afford 36.0 g of the desired thioamide (**2e**) as yellow crystalline solid (Figure S1, c).

2. NMR data of amides 2–6

***N*-Phenyl benzothioamide (2a)**[1] Yield: 85%; Yellow solid, m.p.: 103.6-104.5 °C; ^{1}H NMR (DMSO-d_6, 500 MHz): δ = 11.79 (br, 1H), 7.88 (d, *J* = 7.5 Hz, 4H), 7.54 (t, *J* = 7.0 Hz, 1H), 7.51-7.43 (m, 4H), 7.29 (t, *J* = 7.5 Hz, 1H). ^{13}C NMR (DMSO-d_6, 125 MHz): δ = 197.7, 142.7, 140.1, 130.7, 128.5, 128.0, 127.5, 126.3, 124.2.

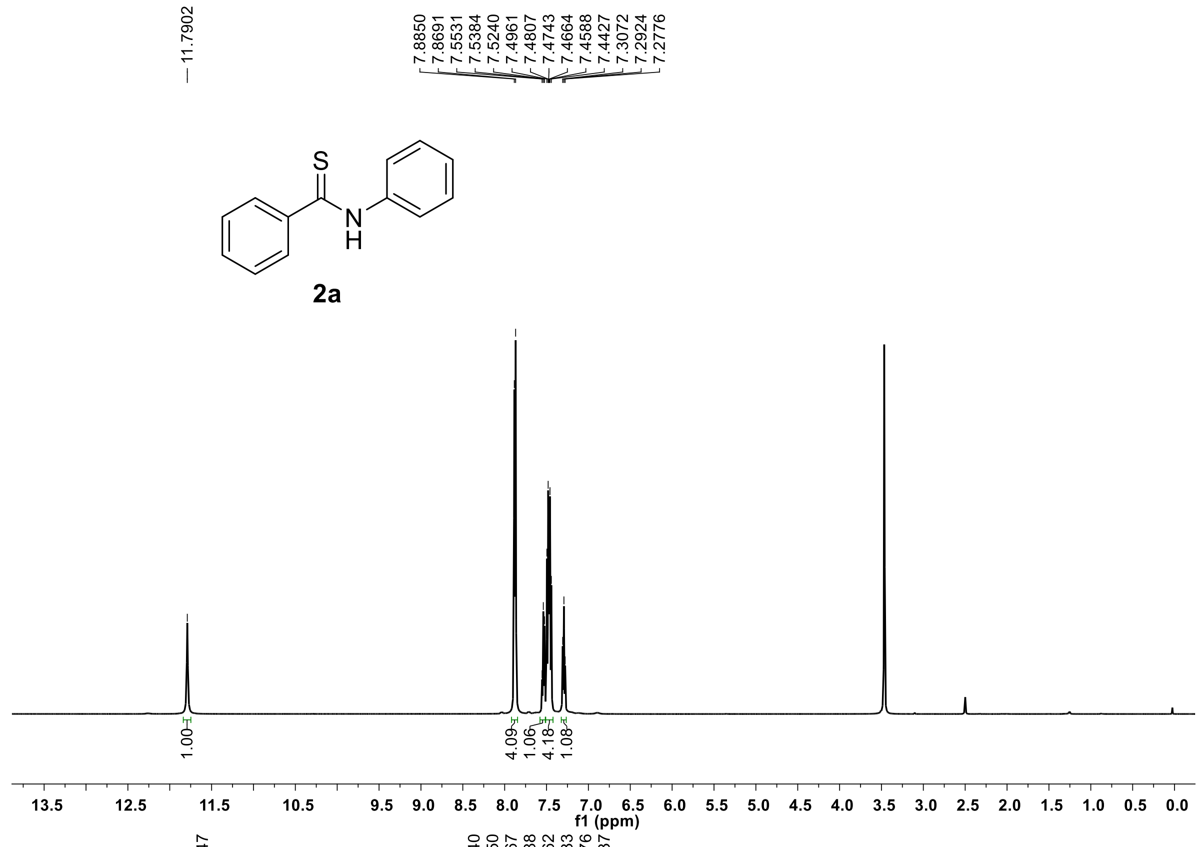

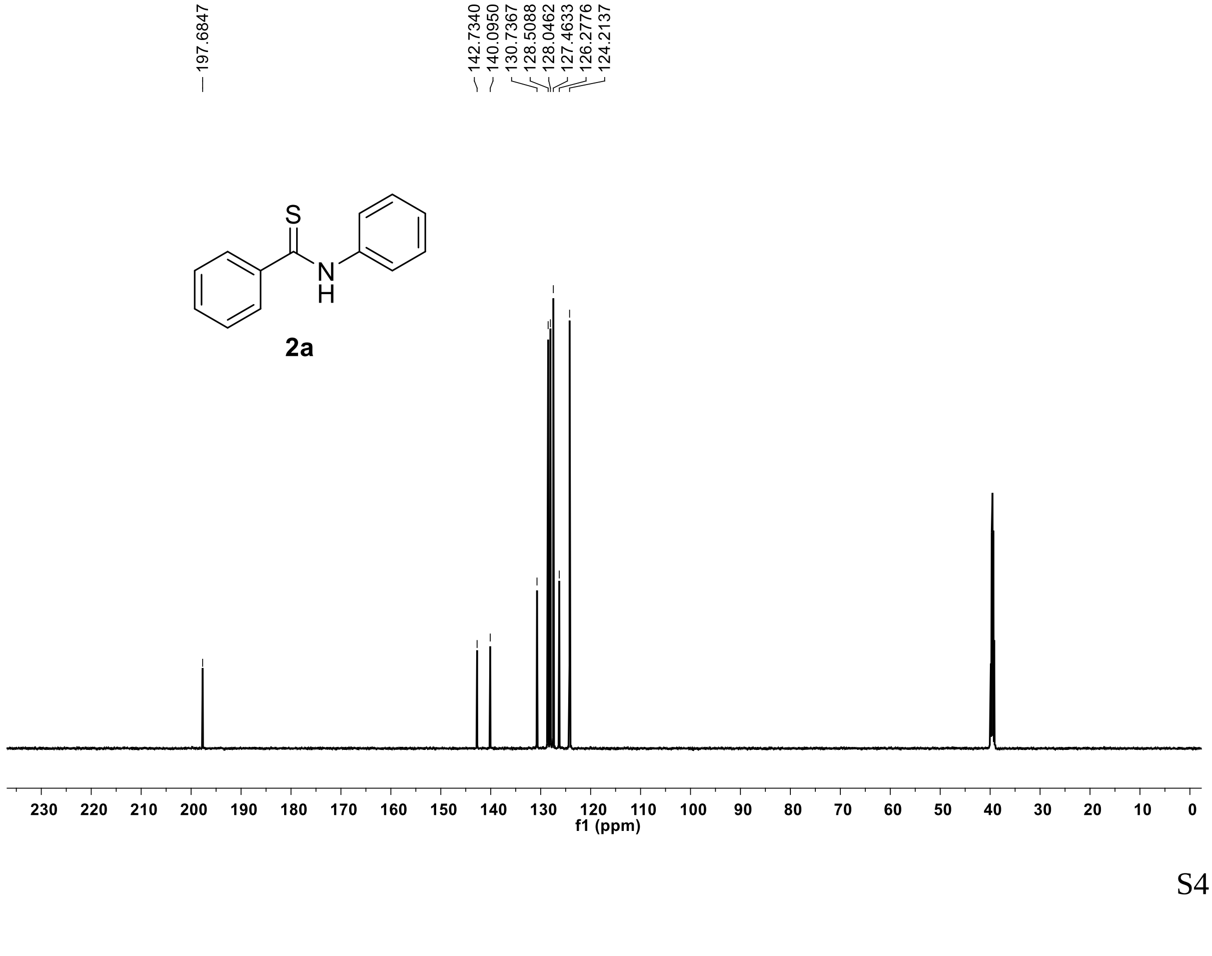

***N*-Phenyl 4-methylbenzothioamide (2b)**[2] Yield: 79%; Yellow solid, m.p.: 128.5-130.2 °C; ^{1}H NMR (DMSO-d_6, 500 MHz): δ = 11.64 (br, 1H), 7.82-7.75 (m, 4H), 7.43 (t, *J* = 7.8 Hz, 2H), 7.30-7.24 (m, 3H), 2.36 (s, 3H). ^{13}C NMR (DMSO-d_6, 125 MHz): δ = 197.3, 140.8, 140.1, 139.8, 128.5, 128.4, 127.5, 126.2, 124.3, 20.9.

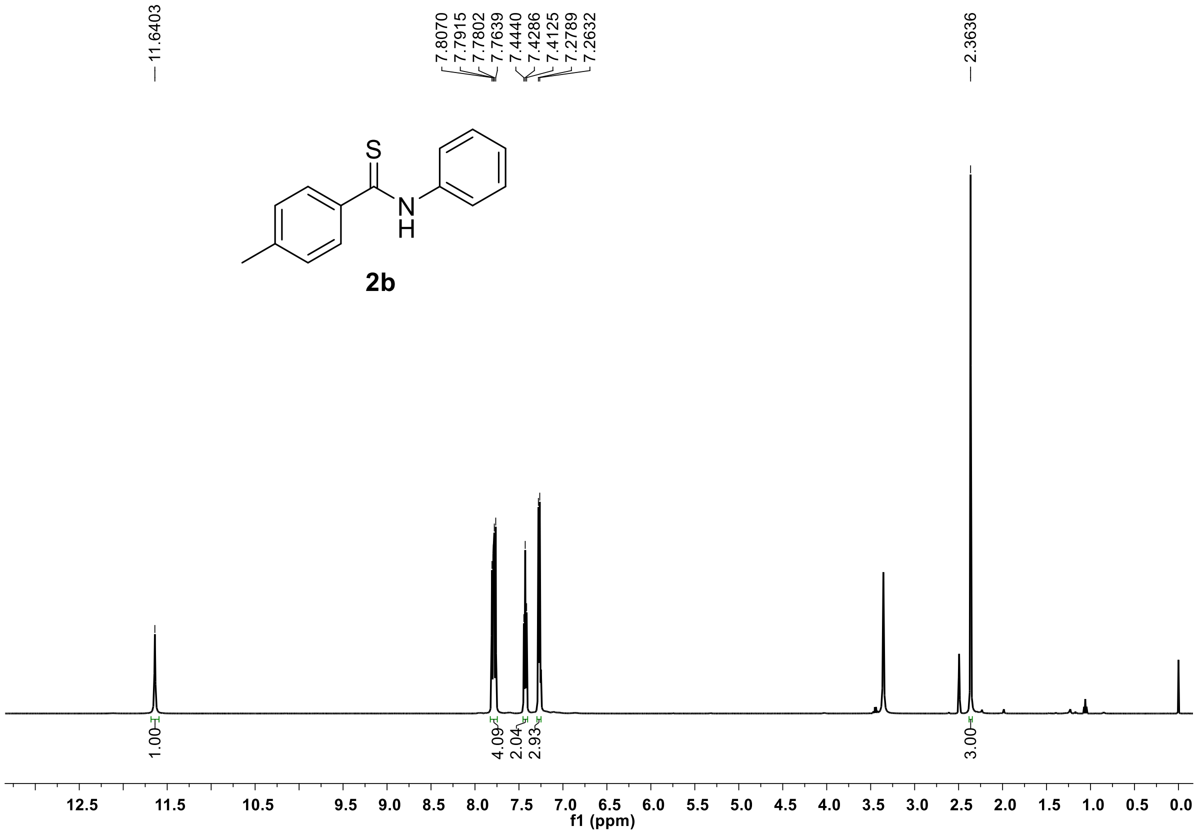

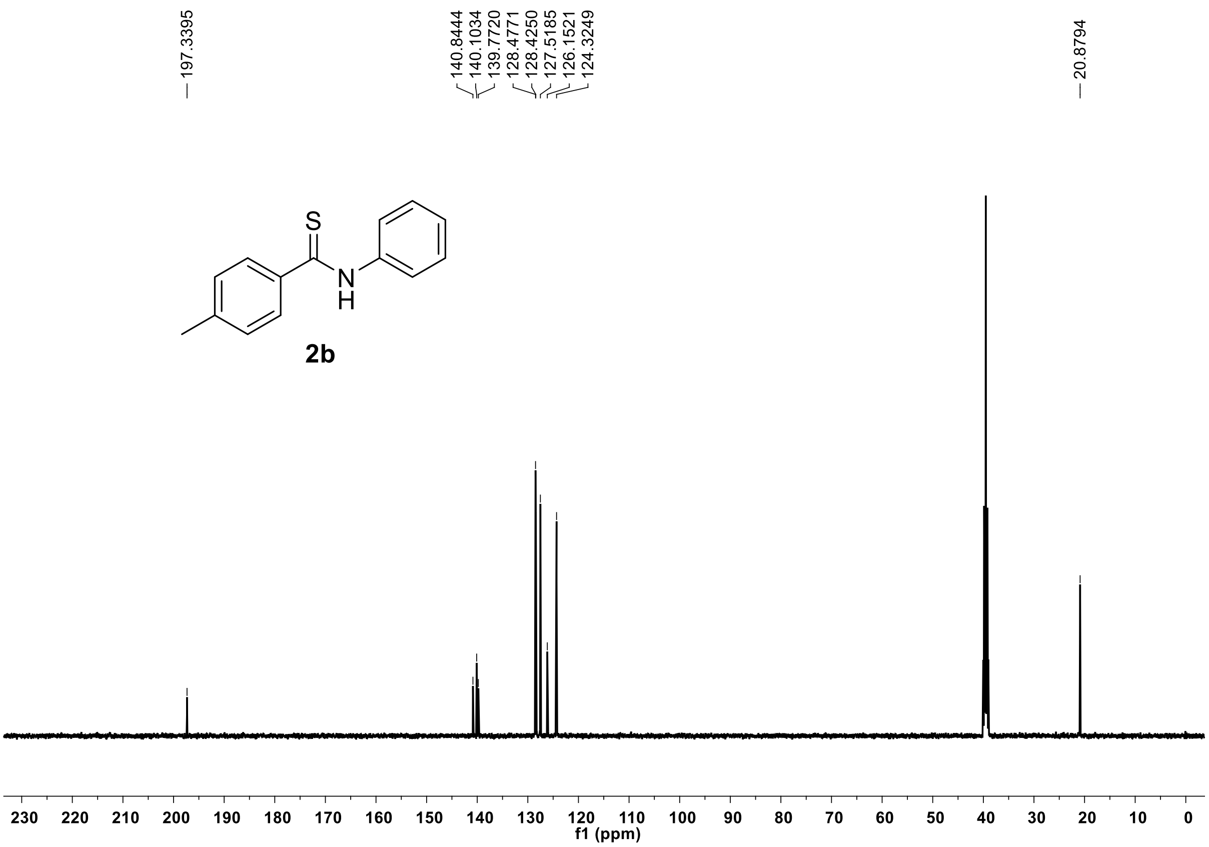

***N*-Phenyl 4-bromobenzothioamide (2c)**[3] Yield: 79%; Yellow solid, m.p.: 128.5-130.3 °C; ^{1}H NMR ($CDCl_3$, 500 MHz): δ = 8.97 (br, 1H), 7.80-7.68 (m, 4H), 7.62-7.53 (m, 2H), 7.49-7.40 (m, 2H), 7.35-7.28 (m, 1H). ^{13}C NMR ($CDCl_3$, 125 MHz): δ = 197.1, 141.9, 139.0, 131.9, 129.3, 128.4, 127.3, 126.1, 123.9.

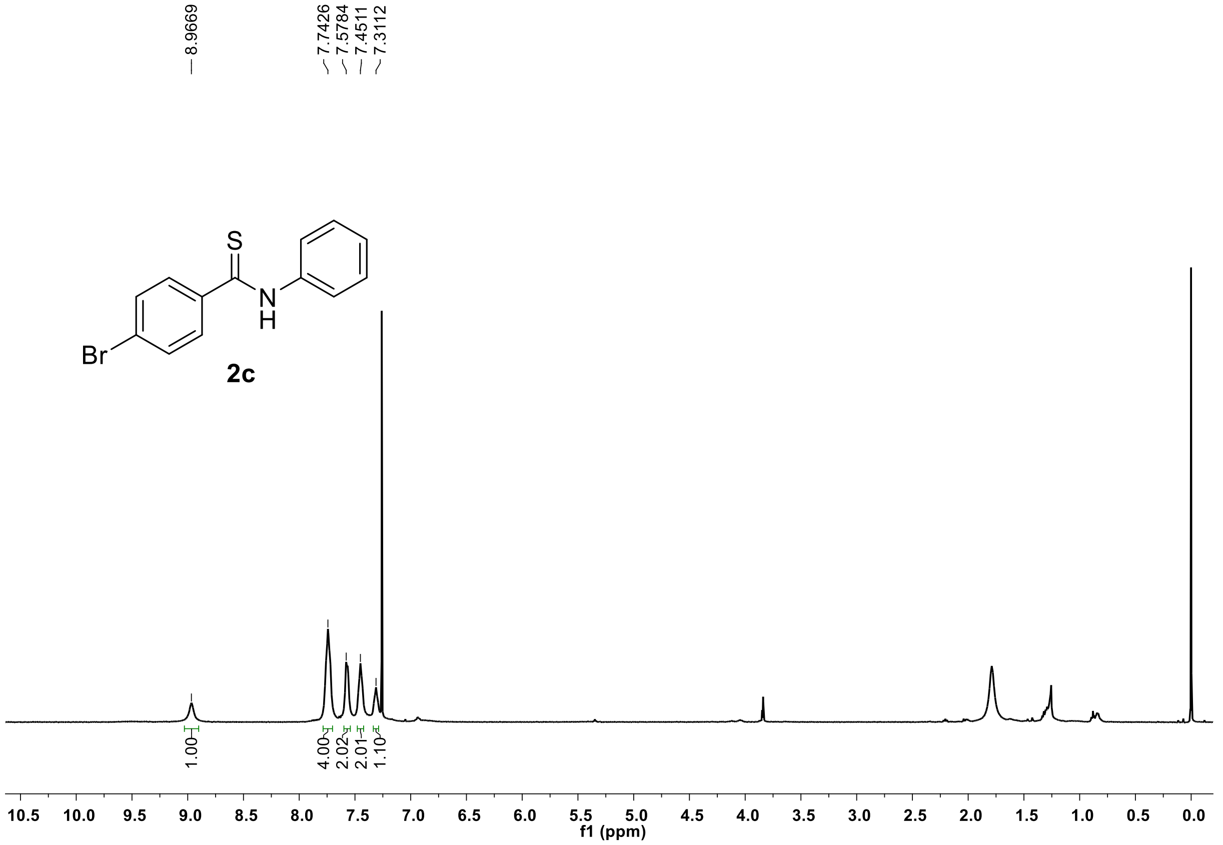

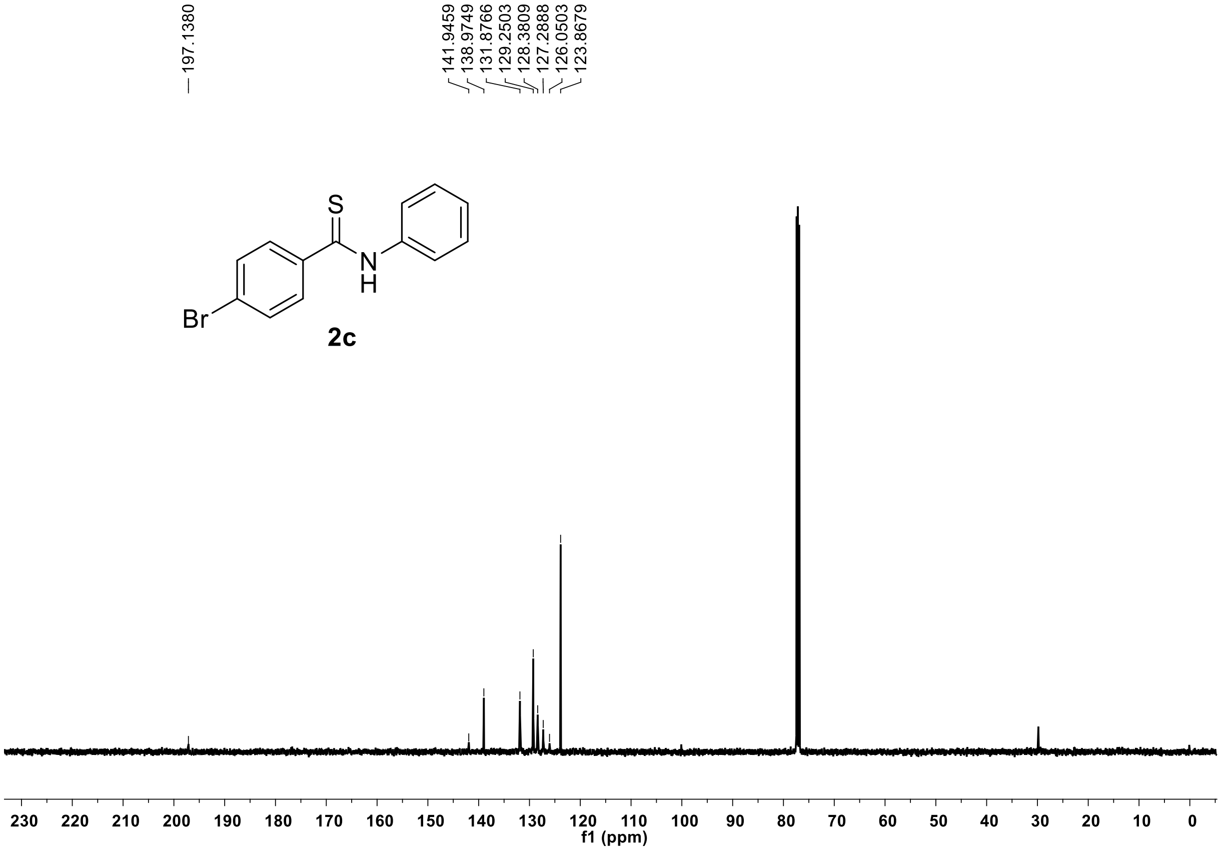

***N*-Phenyl 4-*tert*-butylbenzothioamide (2d)**[4] Yield: 75%; Yellow solid, m.p.: 131.6-132.9 °C; ^{1}H NMR ($CDCl_3$, 500 MHz): δ = 8.99 (br, 1H), 7.87-7.70 (m, 4H), 7.51-7.39 (m, 4H), 7.33-7.27 (m, 1H), 1.35 (s, 9H). ^{13}C NMR ($CDCl_3$, 125 MHz): δ = 198.3, 155.0, 140.2, 139.2, 129.0, 126.9, 126.7, 125.6, 123.9, 35.0, 31.2.

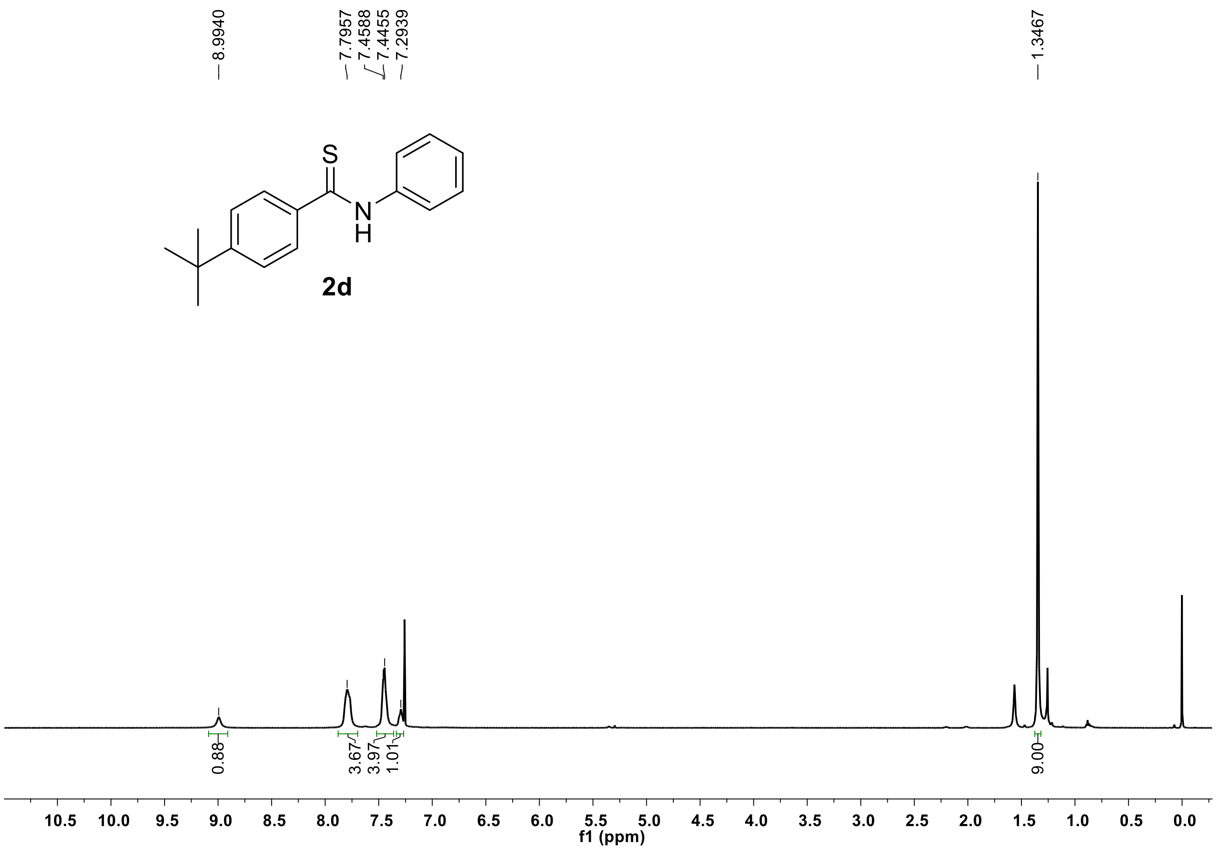

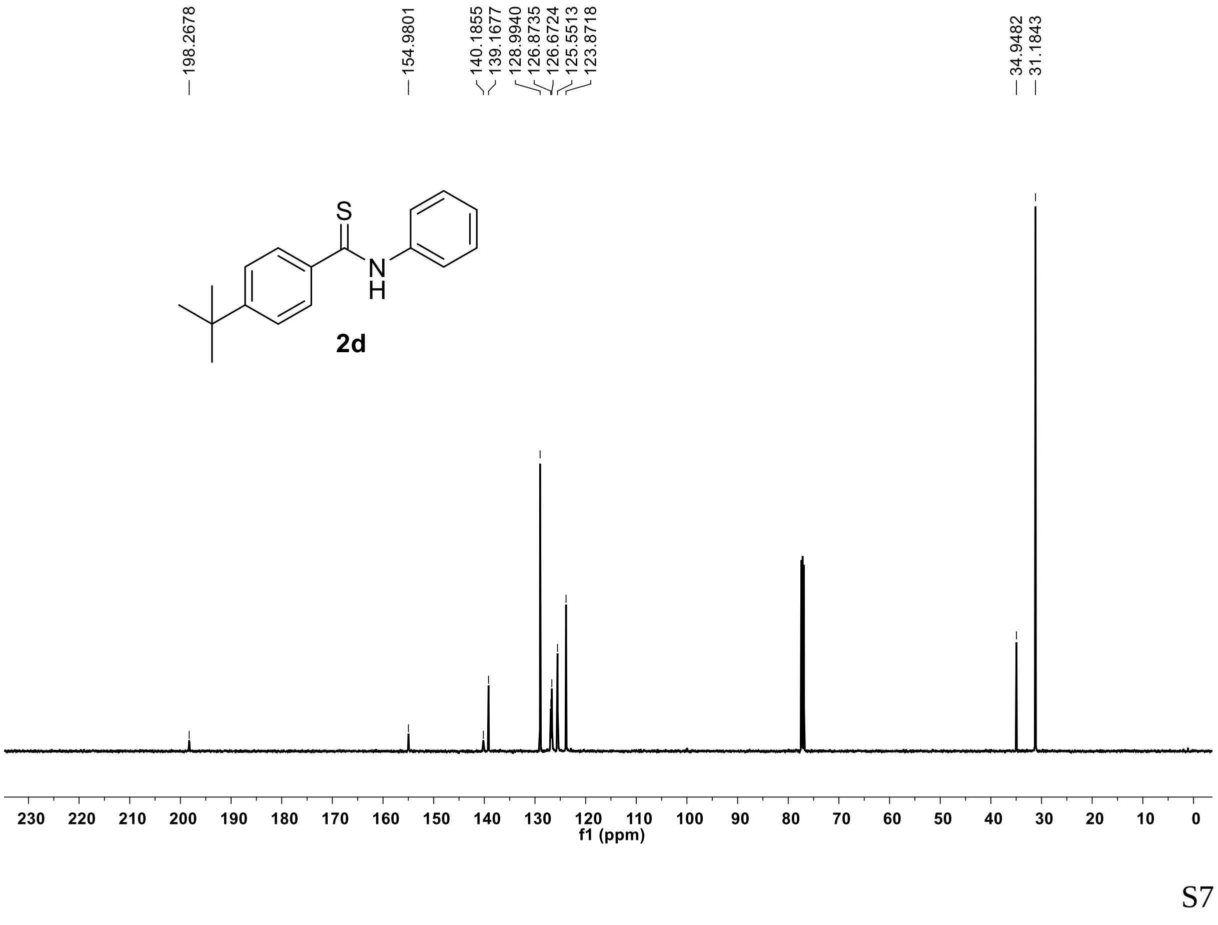

***N*-(4-Methylphenyl) benzothioamide (2e)[1]** Yield: 82%; Yellow solid, m.p.: 128.5-130.1 °C; ^{1}H NMR (DMSO-d_6, 500 MHz): δ = 11.67 (br, 1H), 7.82 (d, *J* = 7.6 Hz, 2H), 7.69 (d, *J* = 7.6 Hz, 2H), 7.52 (t, *J* = 7.0 Hz, 1H), 7.46 (t, *J* = 7.6 Hz, 2H), 7.24 (d, *J* = 7.6 Hz, 2H), 2.32 (s, 3H). ^{13}C NMR (DMSO-d_6, 125 MHz): δ = 197.4, 142.7, 137.6, 135.7, 130.7, 129.0, 128.1, 127.5, 124.2, 20.8.

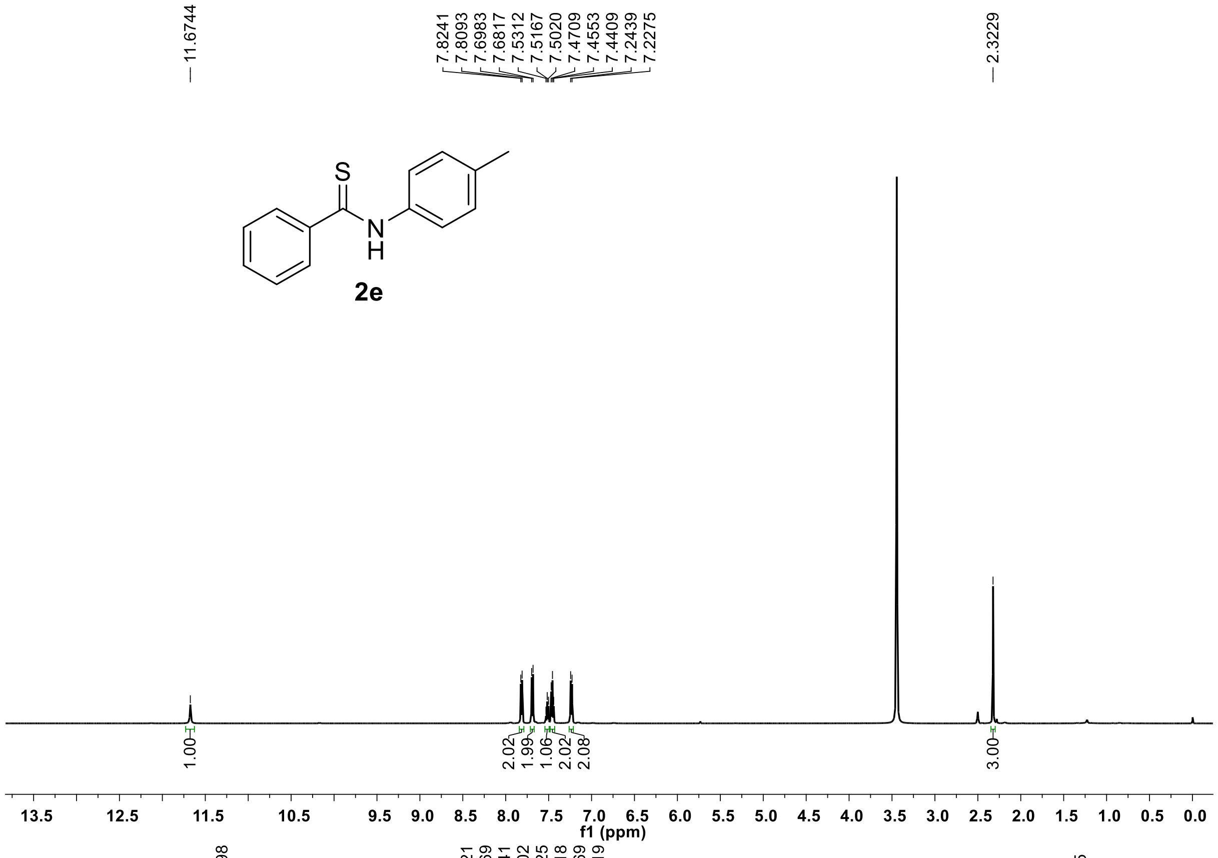

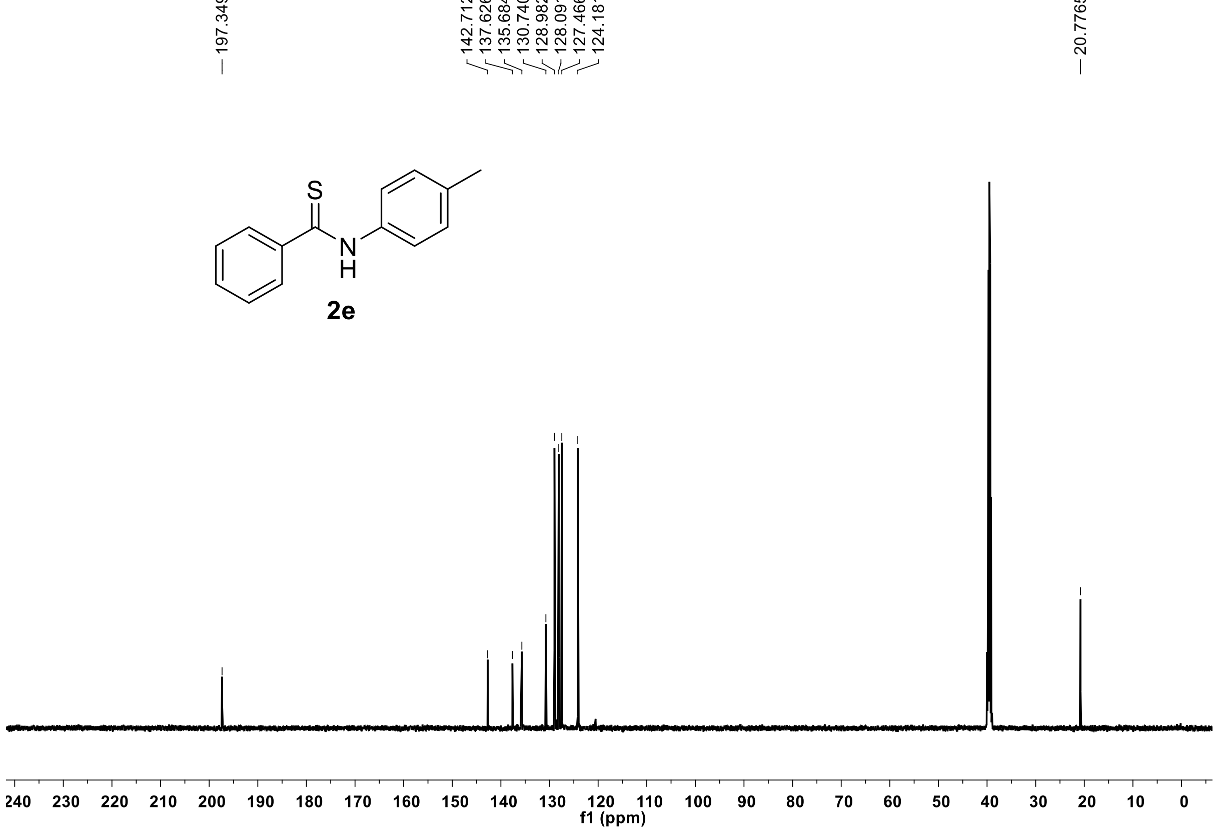

***N*-(4-Chlorophenyl) benzothioamide (2f)**[3] Yield: 92%; Yellow solid, m.p.: 151.5-153 °C; ^{1}H NMR ($CDCl_3$, 400 MHz): δ = 9.00 (br, 1H), 7.92-7.65 (m, 4H), 7.58-7.32 (m, 5H). ^{13}C NMR ($CDCl_3$, 100 MHz): δ = 198.8, 143.0, 137.6, 132.3, 131.6, 129.3, 128.8, 126.8, 125.2.

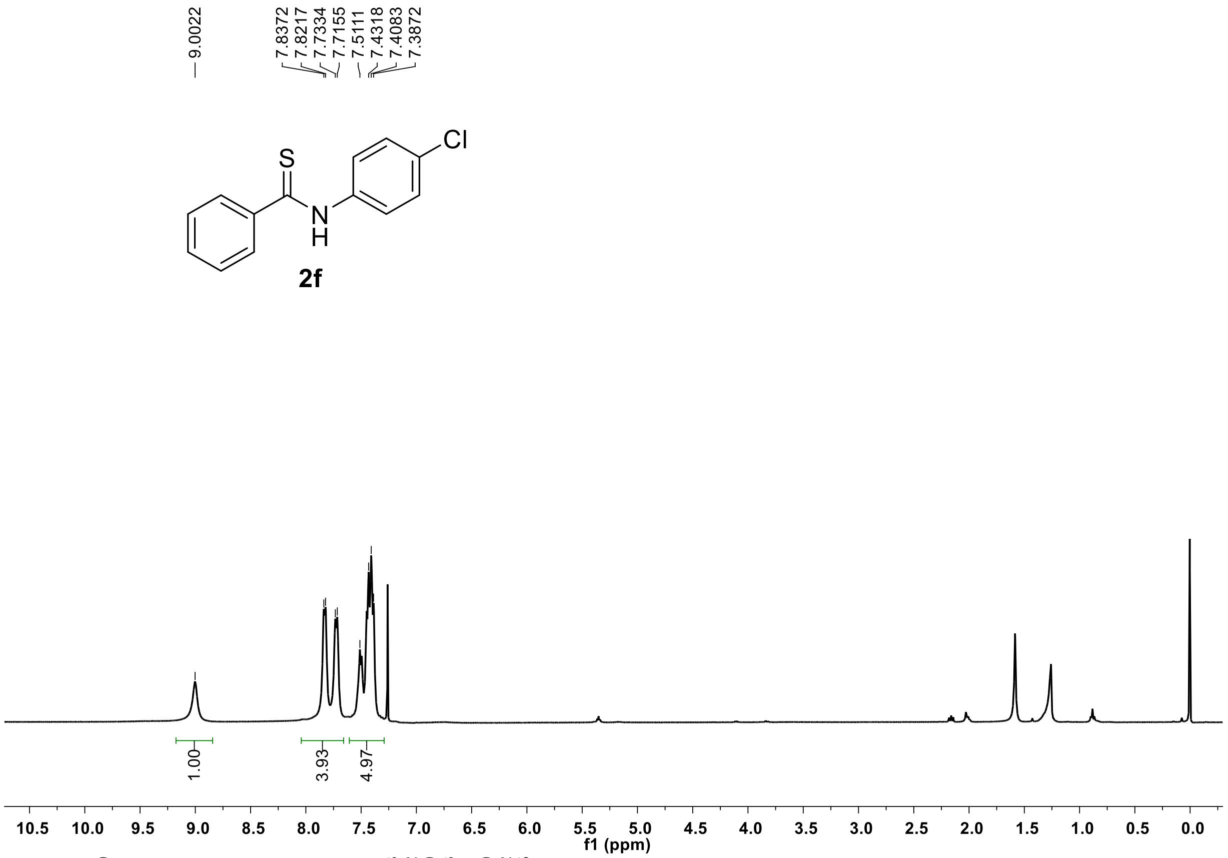

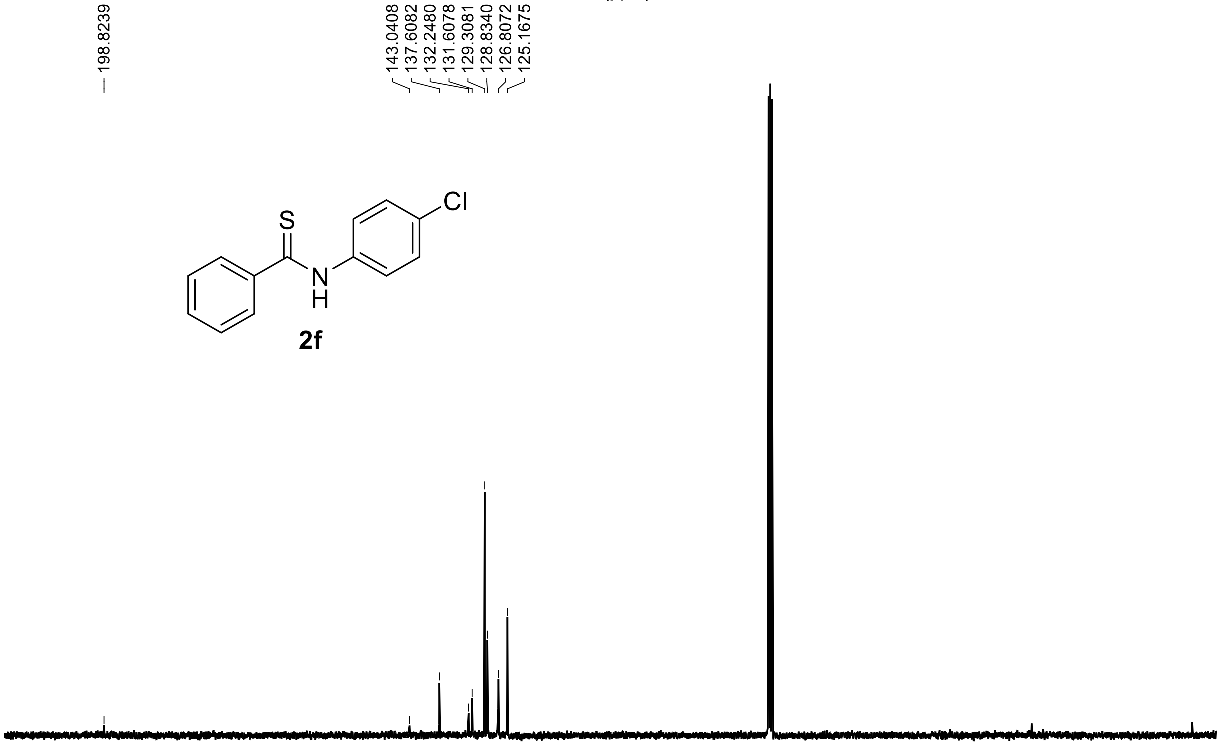

***N*-(4-Bromophenyl) benzothioamide (2g)**[3] Yield: 84%; Yellow solid, m.p.: 148.5-150.3 °C; ^{1}H NMR ($CDCl_3$, 500 MHz): δ = 8.97 (br, 1H), 7.90-7.80 (m, 2H), 7.75-7.64 (m, 2H), 7.60-7.48 (m, 3H), 7.47-7.39 (m, 2H). ^{13}C NMR ($CDCl_3$, 125 MHz): δ = 198.7, 143.0, 138.1, 132.2, 131.6, 128.8, 126.8, 125.4, 120.0.

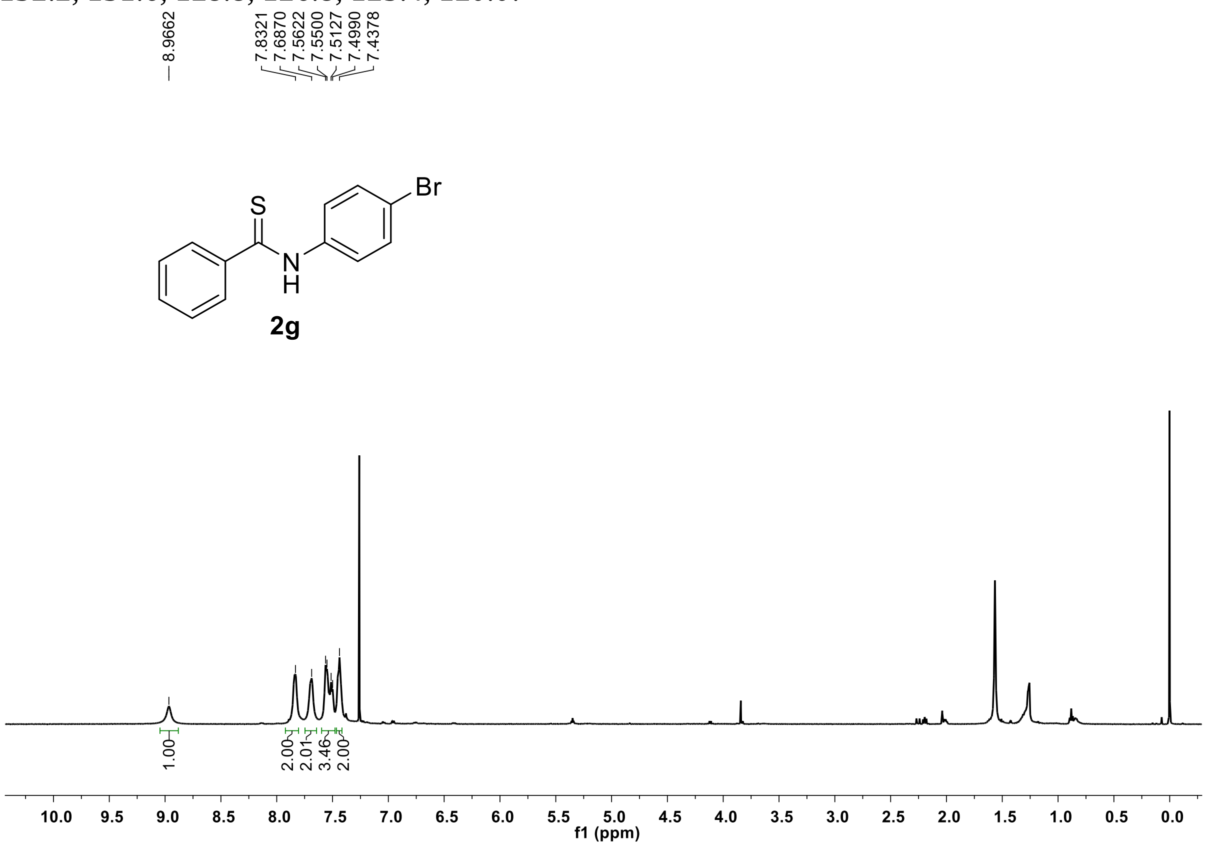

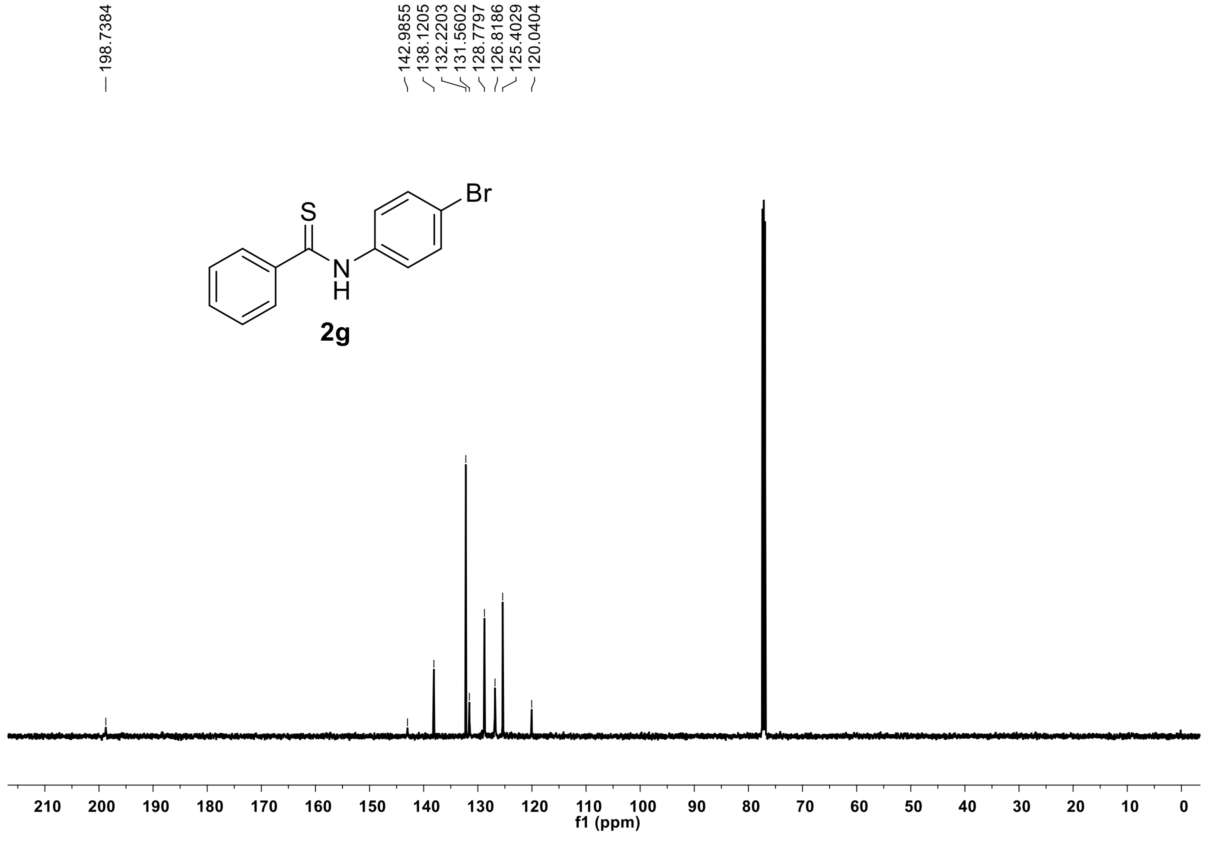

***N*-(4-Iodophenyl) benzothioamide (2h)**[5] Yield: 78%; Yellow solid, m.p.: 143.6-145.9 °C; ^{1}H NMR (DMSO-d_6, 500 MHz): δ = 11.79 (br, 1H), 7.84 (d, *J* = 7.5 Hz, 2H), 7.79 (d, *J* = 8.0 Hz, 2H), 7.72 (d, *J* = 8.0 Hz, 2H), 7.56-7.50 (m, 1H), 7.50-7.42 (m, 2H). ^{13}C NMR (DMSO-d_6, 125 MHz): δ = 197.7, 142.6, 139.8, 137.2, 130.8, 128.0, 127.4, 126.1, 90.9.

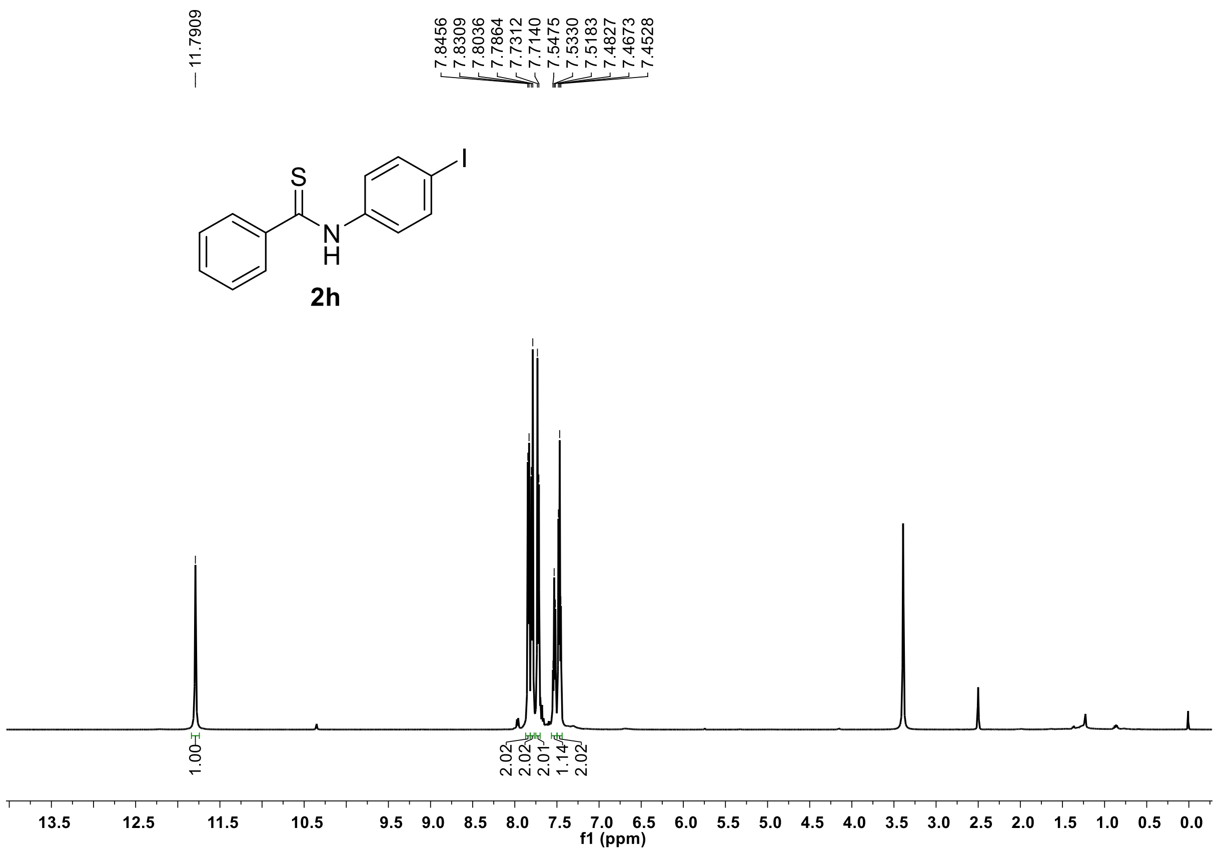

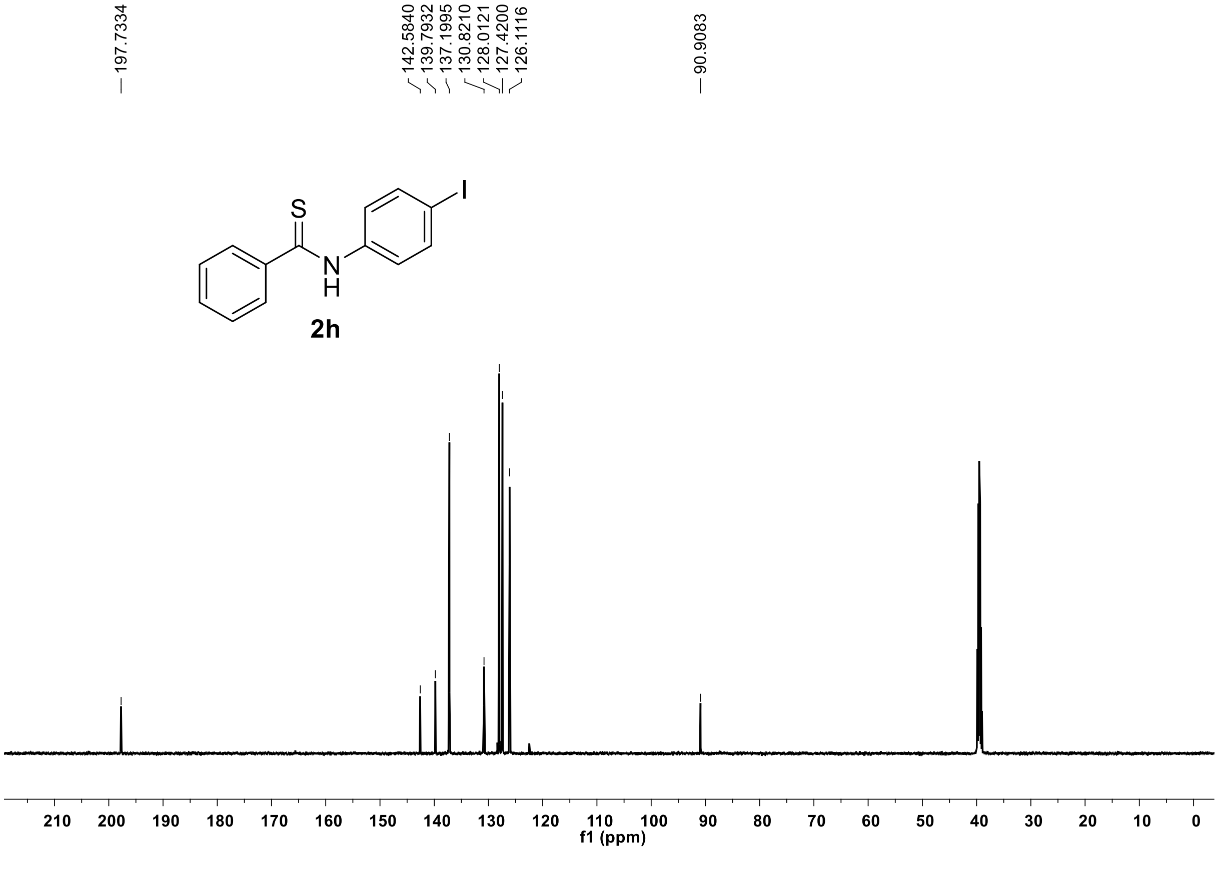

***N*-(3-Chlorophenyl) benzothioamide (2i)**[3] Yield: 75%; Yellow solid, m.p.: 96.5-97.8 °C; ^{1}H NMR (DMSO-d_6, 500 MHz): δ = 11.86 (br, 1H), 8.08 (s, 1H), 7.88-7.78 (m, 3H), 7.54 (t, *J* = 7.1 Hz, 1H), 7.51-7.45 (m, 3H), 7.35 (d, *J* = 8.0 Hz, 1H); ^{13}C NMR (DMSO-d_6, 125 MHz): δ = 198.2, 142.6, 141.4, 132.6, 130.9, 130.2, 128.1, 127.5, 126.0, 123.6, 122.6.

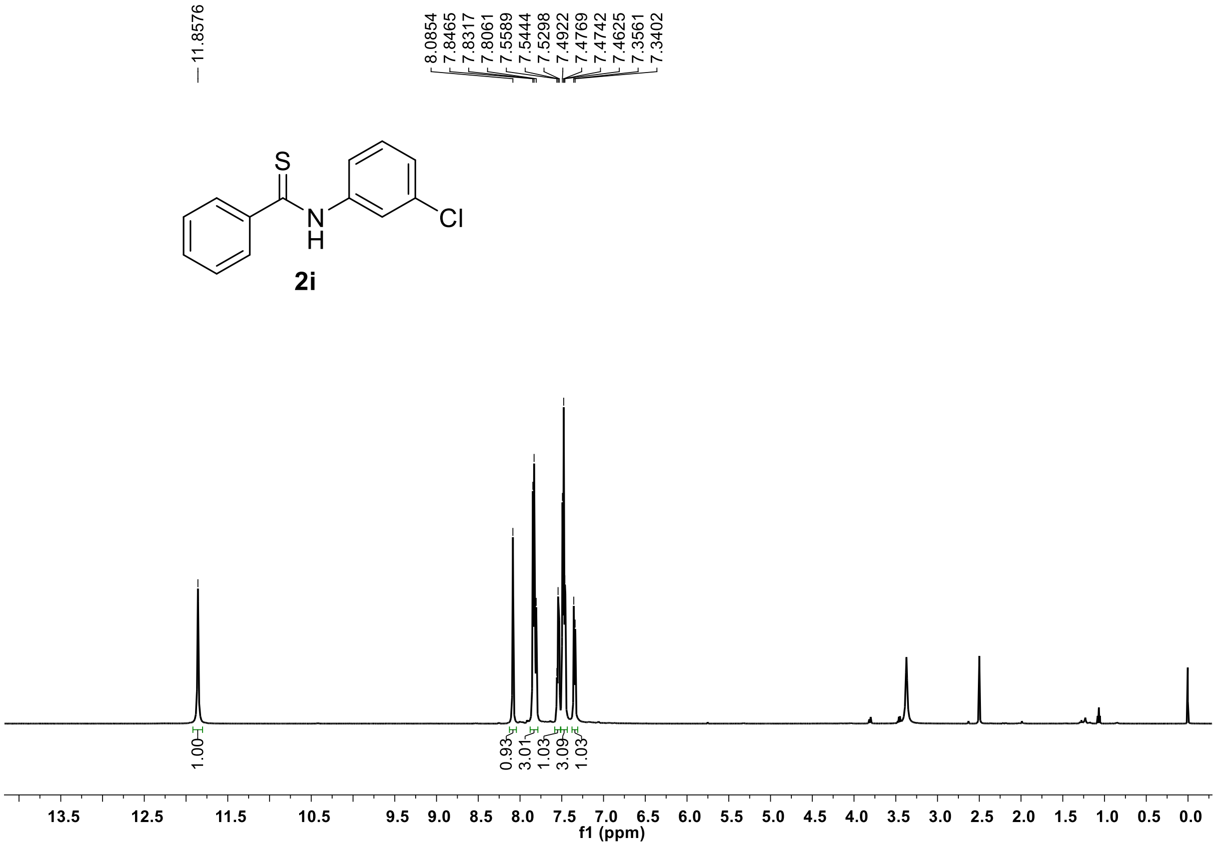

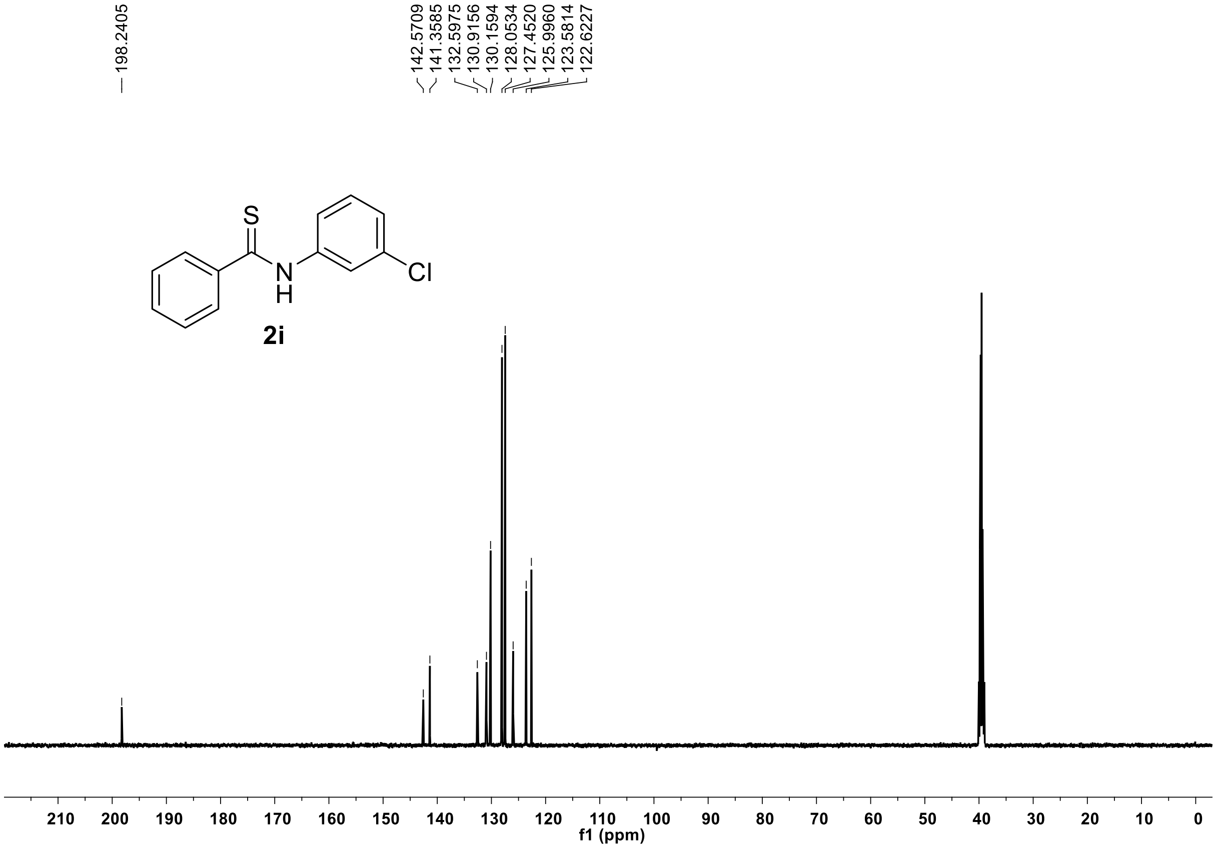

***N*-Phenyl phenylacetothioamide (2j)**[6] Yield: 70%; Yellow solid, m.p.: 88.5-89.1 °C; ^{1}H NMR ($CDCl_3$, 500 MHz): δ = 8.43 (br, 1H), 7.55 (d, *J* = 7.7 Hz, 2H), 7.46-7.41 (m, 2H), 7.39-7.33 (m, 5H), 7.26-7.20 (m, 1H), 4.29 (s, 2H). ^{13}C NMR ($CDCl_3$, 125 MHz): δ = 201.3, 138.6, 135.0, 129.8, 129.6, 129.0, 128.3, 127.2, 123.8, 55.1.

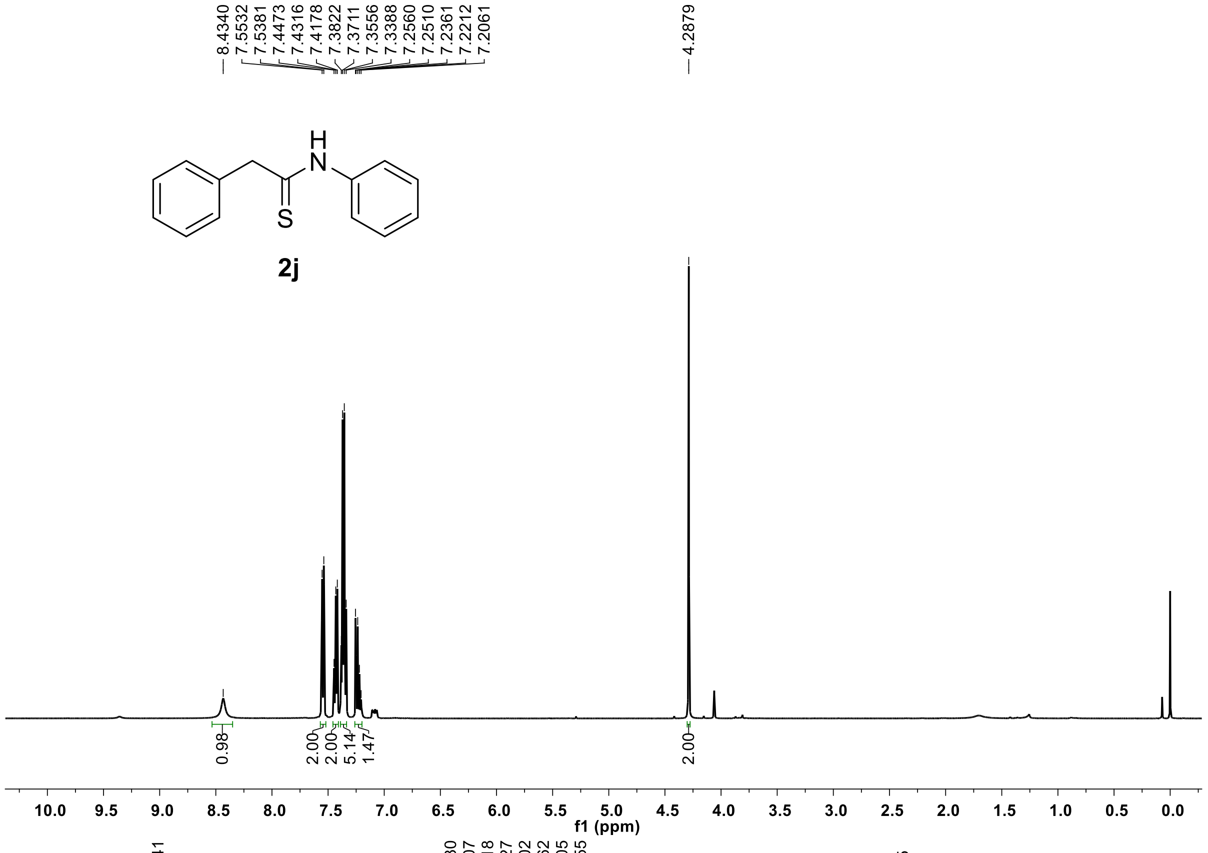

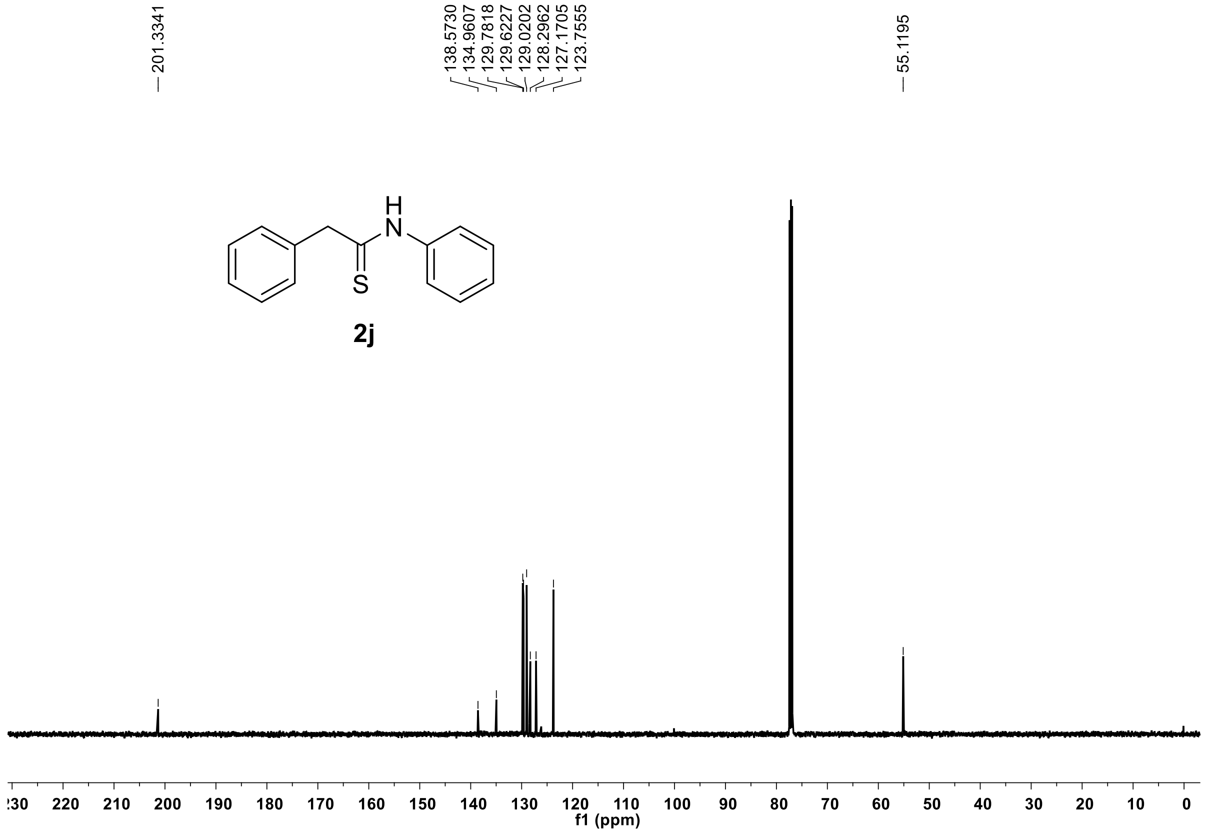

***N*-Phenyl pivalthioamide (2k)**[7] Yield: 76%; Yellow solid, m.p.: 86.5-88.3 °C; ^{1}H NMR ($CDCl_3$, 500 MHz): δ = 8.75 (br, 1H), 7.58 (d, *J* = 7.7 Hz, 2H), 7.42 (t, *J* = 7.5 Hz, 2H), 7.28 (t, *J* = 7.5 Hz, 1H), 1.49 (s, 9H). ^{13}C NMR ($CDCl_3$, 125 MHz): δ = 213.7, 129.1, 127.7, 127.2, 124.9, 45.6, 30.5.

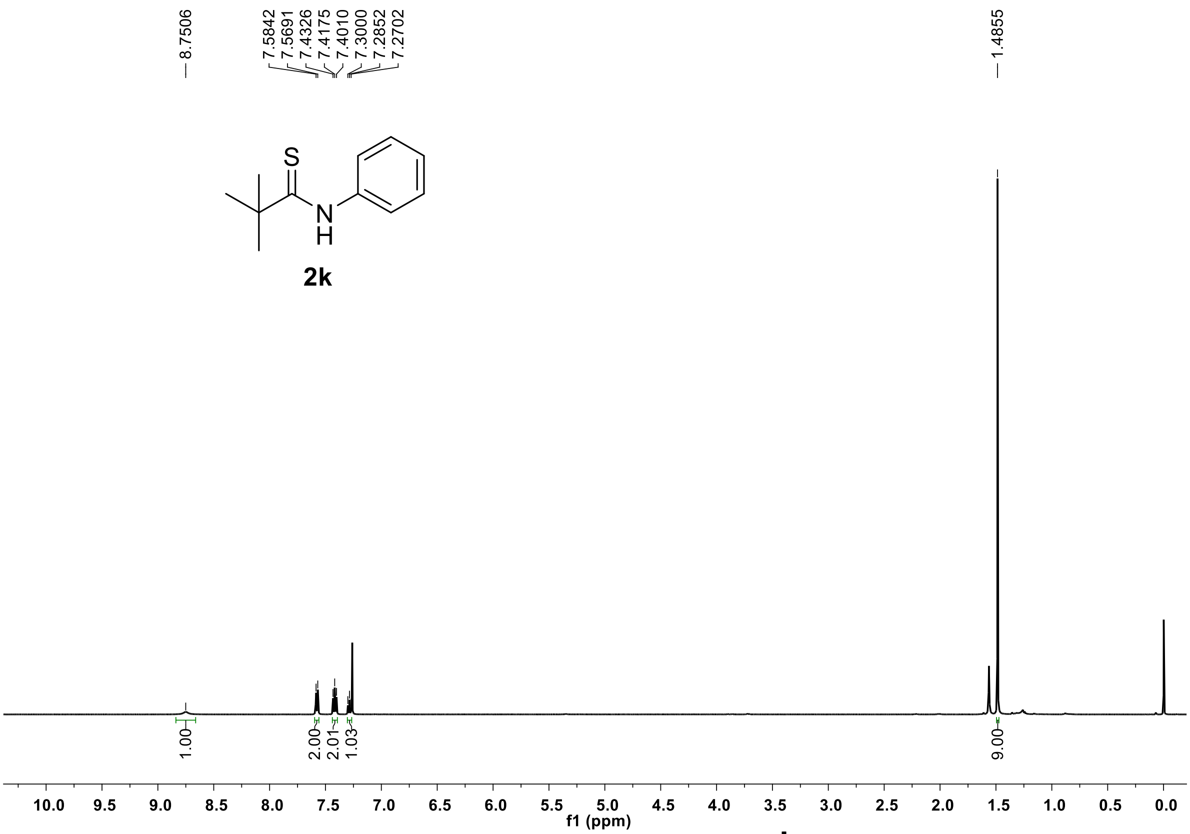

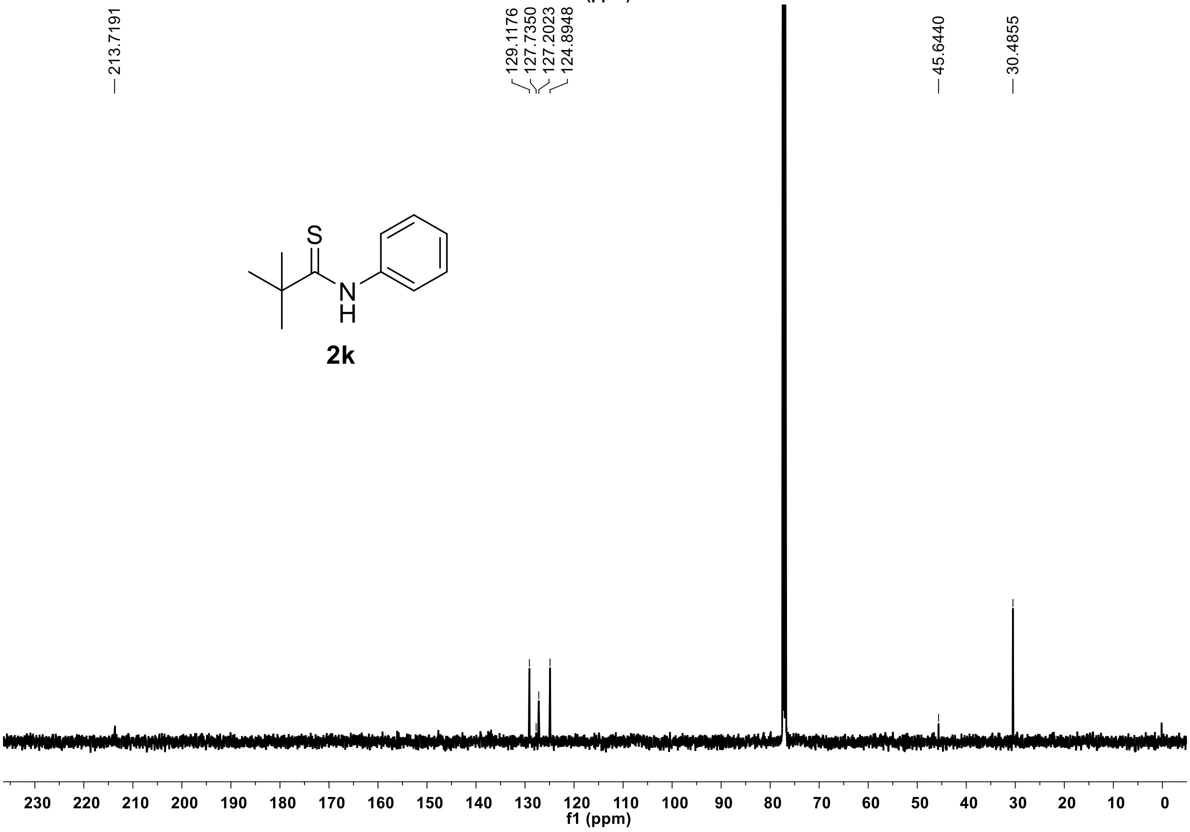

N^2,N^6-Di(*n*-butyl)pyridine-2,6-(carbothioamide) (3)[8] Yield: 70%; White solid. M.p.: 155.2-158.0 °C; ^{1}H NMR ($CDCl_3$, 600 MHz): δ = 8.33 (d, *J* = 7.8 Hz, 2H), 8.03-7.94 (m, 3H), 3.48-3.42 (m, 4H), 1.63-1.55 (m, 4H), 1.42-1.32 (m, 4H), 0.93-0.89 (m, 6H). ^{13}C NMR (150 MHz, $CDCl_3$) δ = 163.7, 149.1, 139.1, 125.0, 39.5, 31.9, 20.3, 13.9.

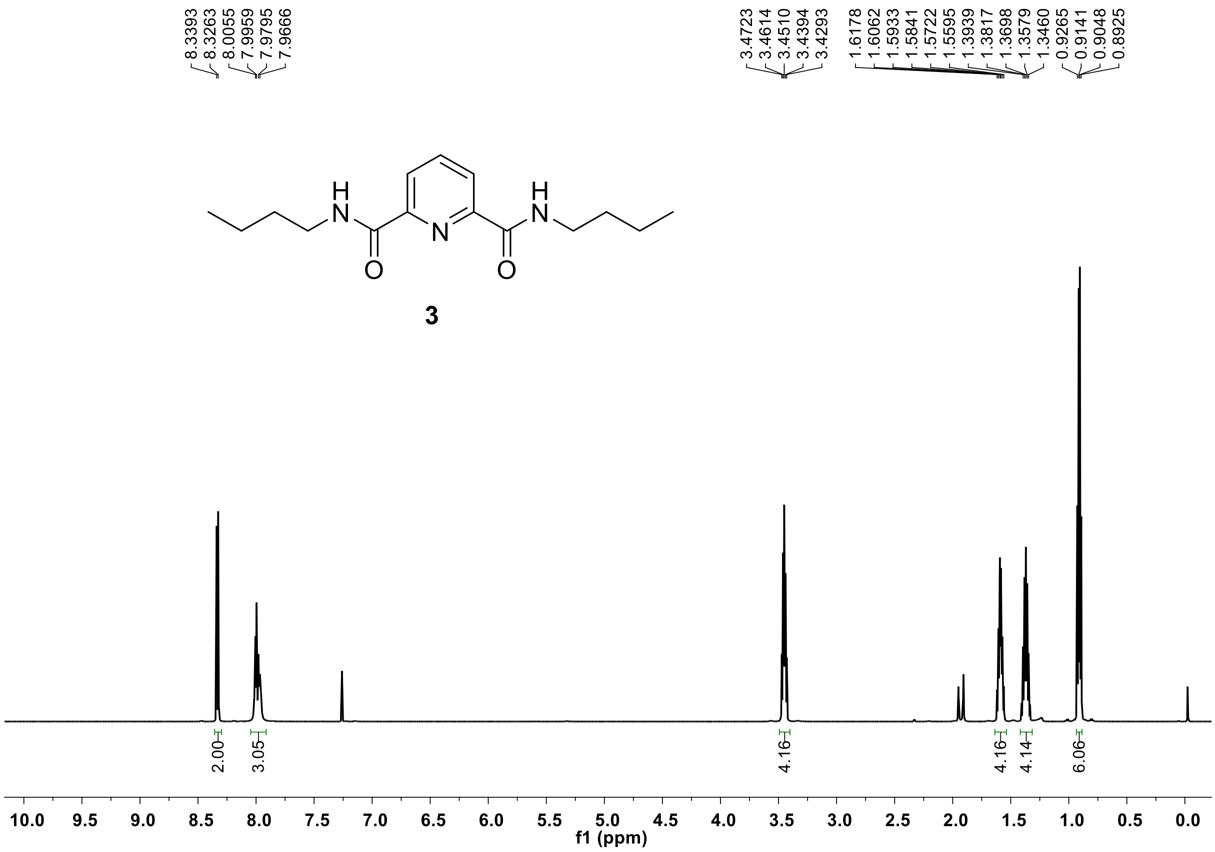

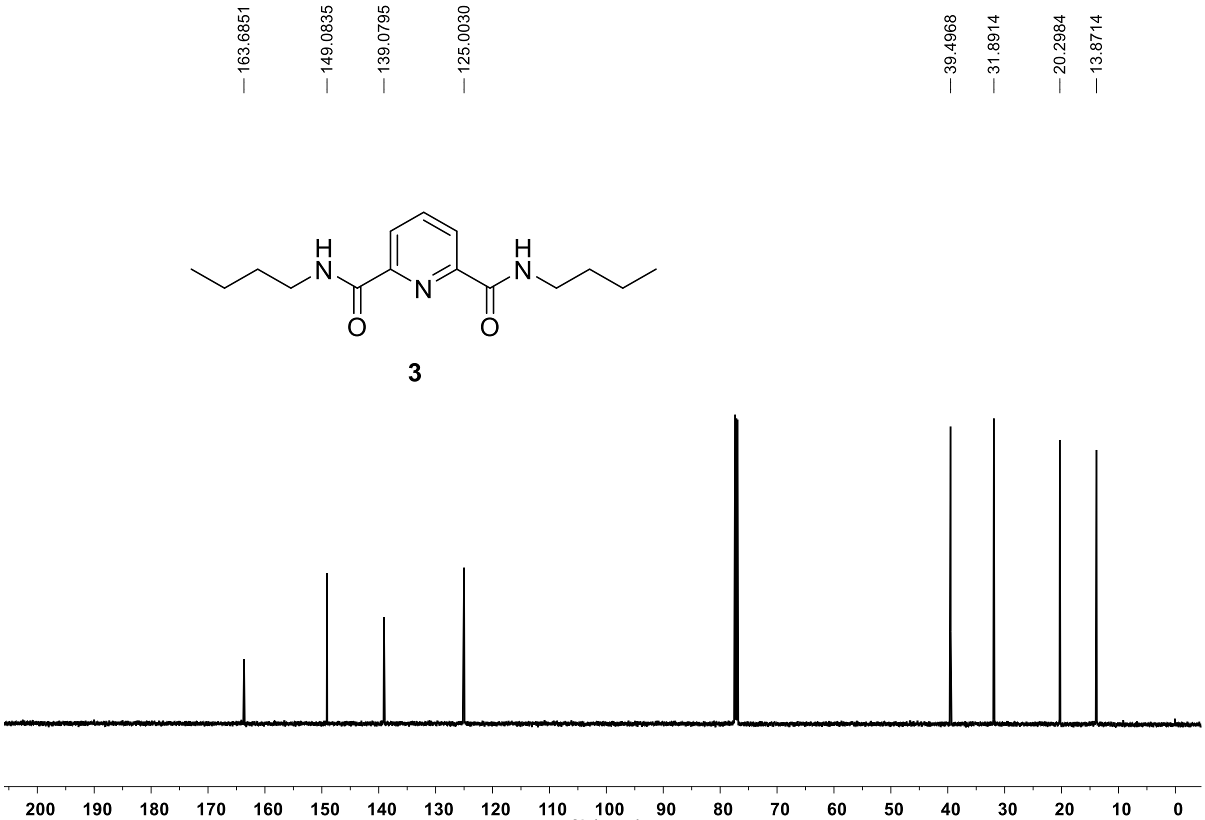

N^2,N^6-Di(*n*-butyl)pyridine-2,6-bis(carbothioamide) (4)[8] Yield: 84%; Yellow solid, m.p.: 72.2-74.5 °C; ^{1}H NMR ($CDCl_3$, 600 MHz): δ = 9.44 (br, 2H), 8.78 (d, *J* = 7.8 Hz, 2 H), 7.94 (t, *J* = 7.8 Hz, 1H), 3.91-3.86 (m, 4H), 1.84-1.77 (m, 4H), 1.55-1.48 (m, 4H), 1.02 (t, *J* = 7.4 Hz, 6H). ^{13}C NMR (150 MHz, $CDCl_3$) δ = 190.4, 149.6, 138.4, 127.3, 45.9, 30.2, 20.5, 13.9.

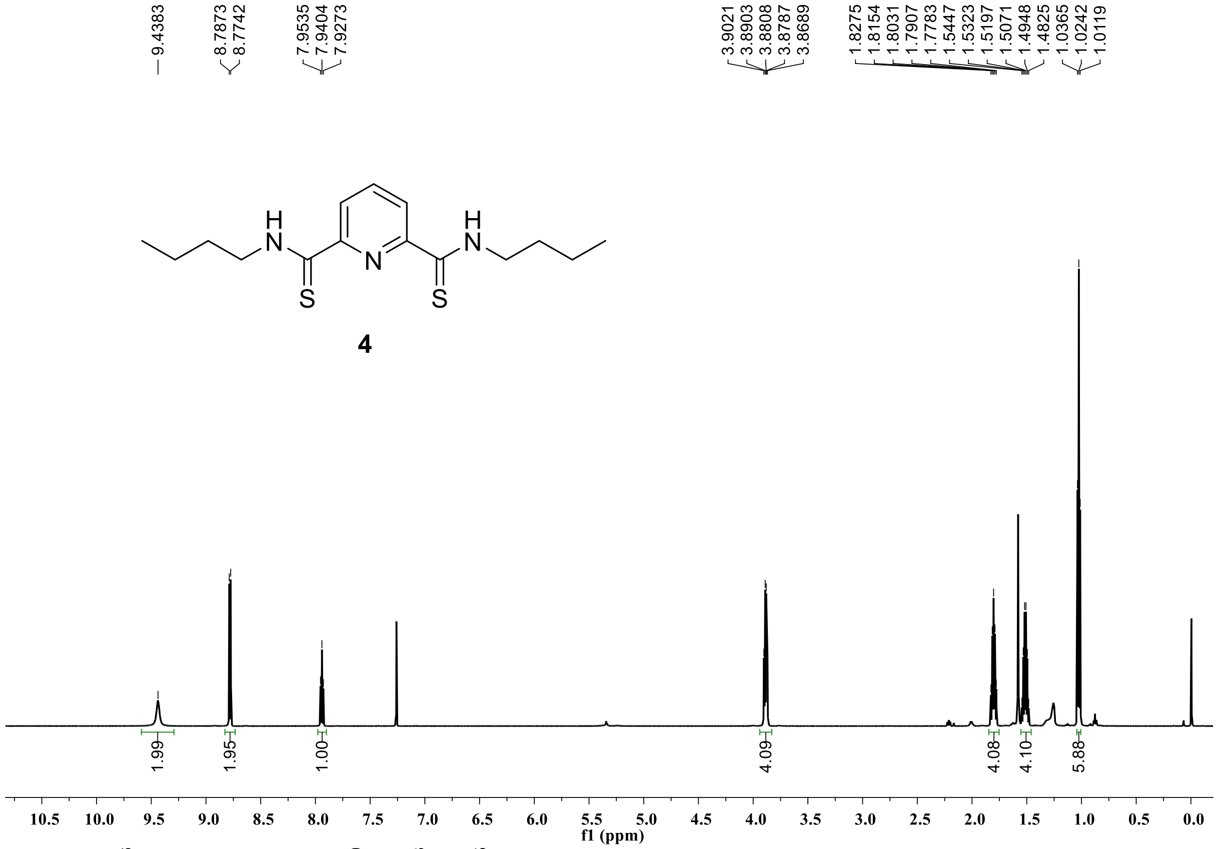

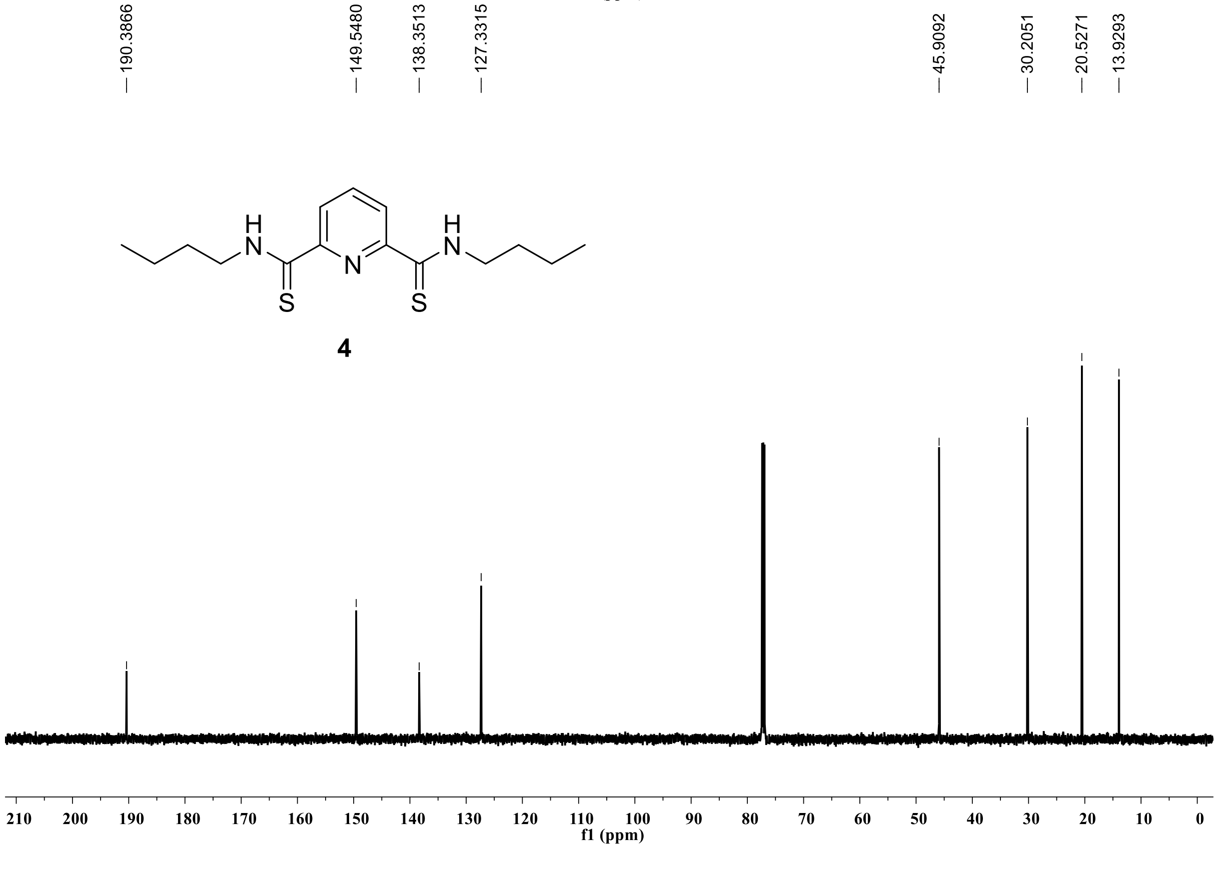

***N*^{2},*N*^{6}-Bis(2,4,6-trimethylphenyl)pyridine-2,6-(carbothioamide) (5)**[8] Yield: 92%; White solid. M.p.: 191.5-193.3 °C; ^{1}H NMR ($CDCl_3$, 600 MHz): δ = 9.05 (s, 1H), 8.51 (d, *J* = 7.8 Hz, 2H), 8.15 (t, *J* = 7.8 Hz, 1H), 6.96 (s, 4H), 2.31 (s, 6H), 2.26 (s, 12 H). ^{13}C NMR (150 MHz, $CDCl_3$) δ = 161.8, 149.0, 139.5, 137.3, 135.1, 130.7, 129.2, 125.7, 77.4, 77.2, 77.0, 21.1, 18.5.

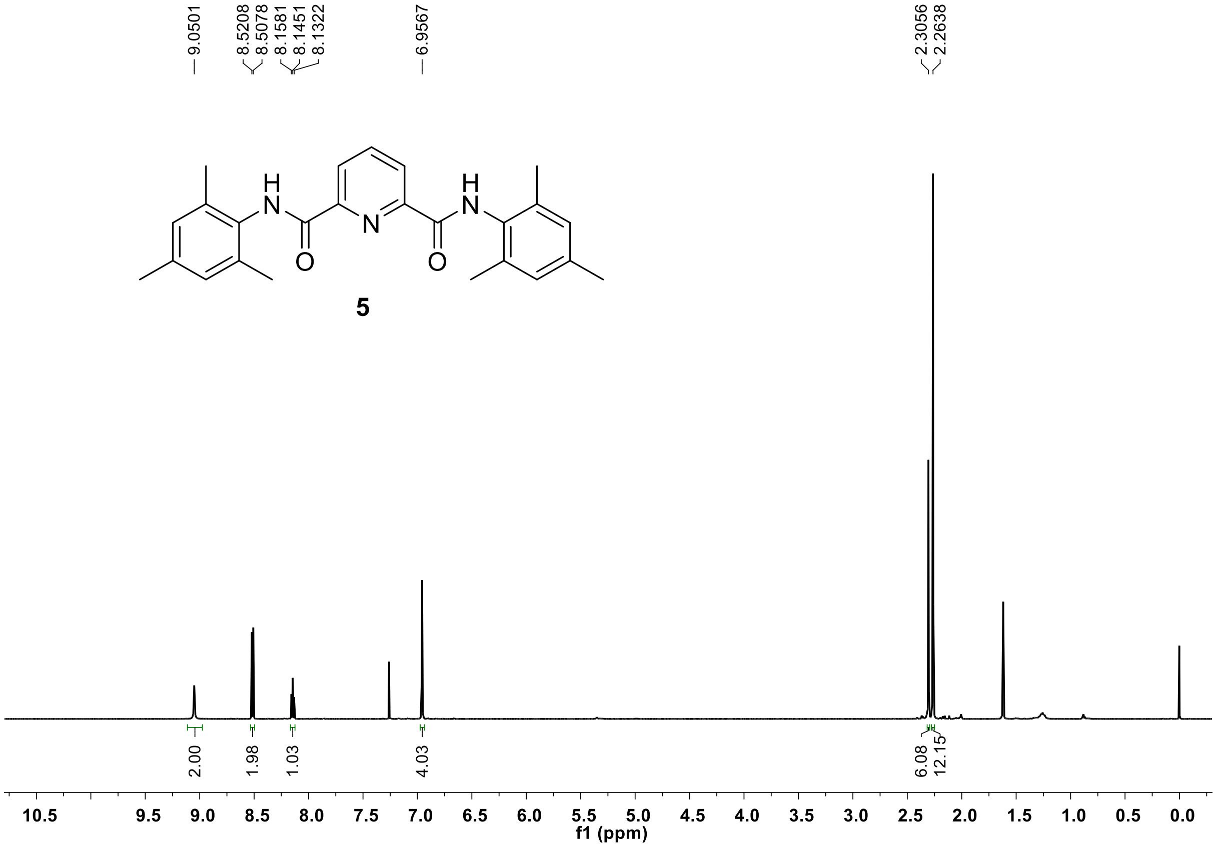

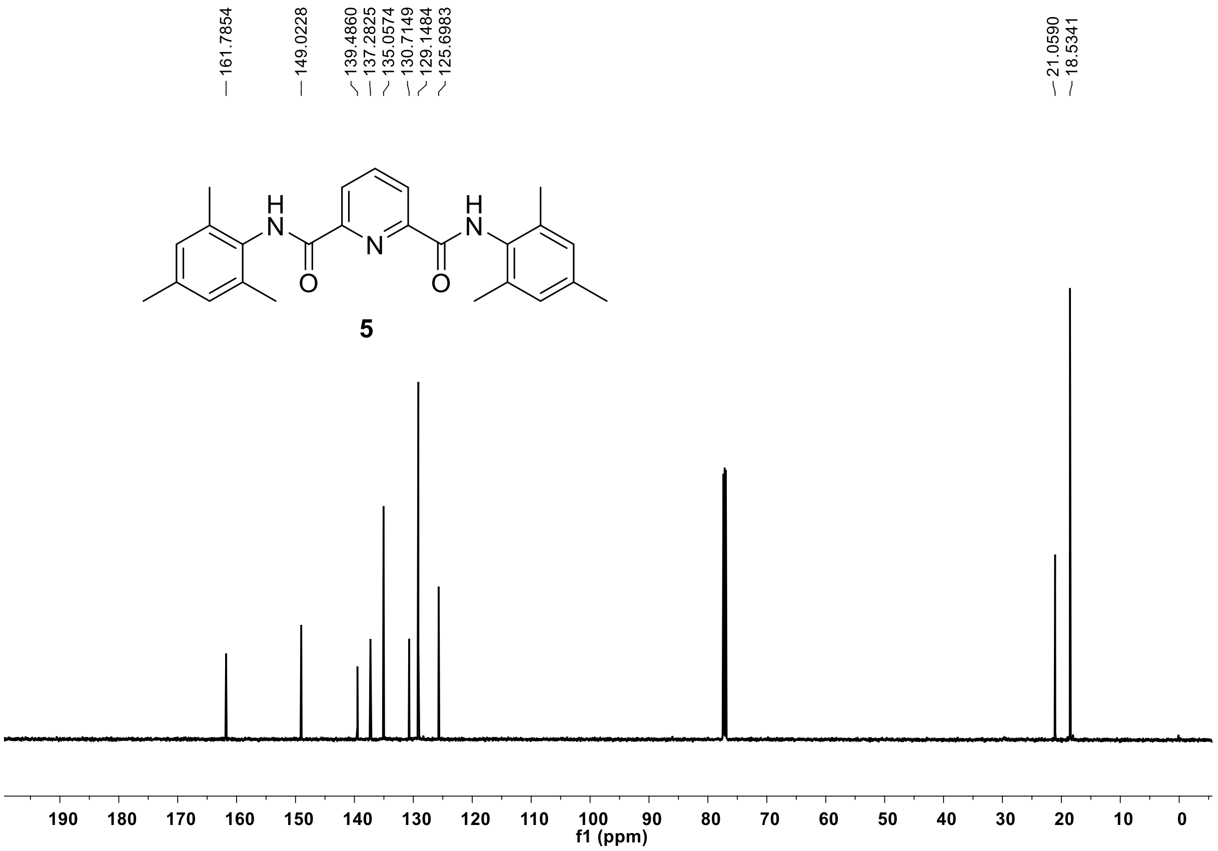

***N*²,*N*⁶-Bis(2,4,6-trimethylphenyl)pyridine-2,6-bis(carbothioamide) (6)**[8] Yield: 91%; Yellow solid, m.p.: 190.5-193.6 °C; ^{1}H NMR ($CDCl_3$, 600 MHz): δ = 10.71 (br, 2H), 8.99 (d, *J* = 7.8 Hz, 2H), 8.07 (t, J = 7.8 Hz, 1H), 6.99 (s, 4H), 2.33 (s, 6H), 2.23 (s, 12 H).^{13}C NMR (150 MHz, $CDCl_3$) δ = 190.8, 149.4, 138.7, 138.5, 135.1, 133.4, 129.4, 128.0, 21.3, 18.3.

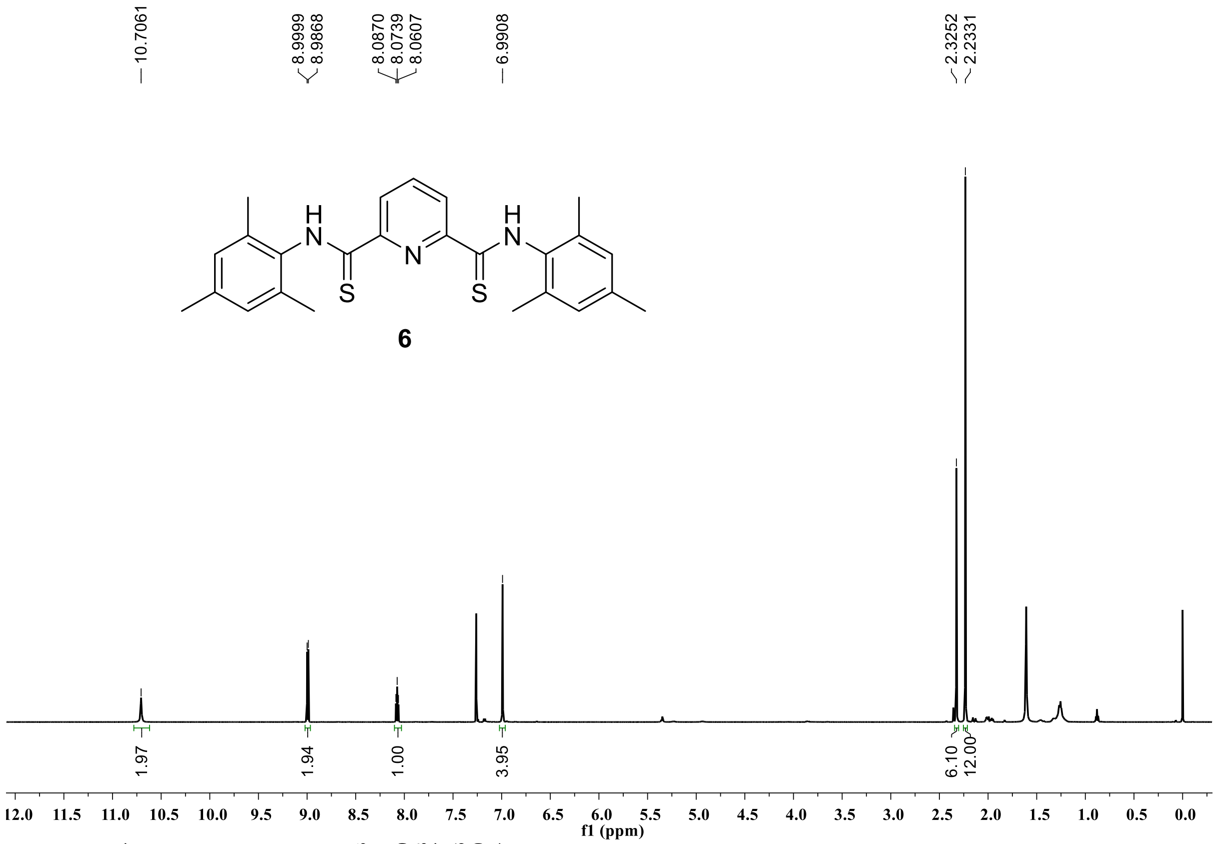

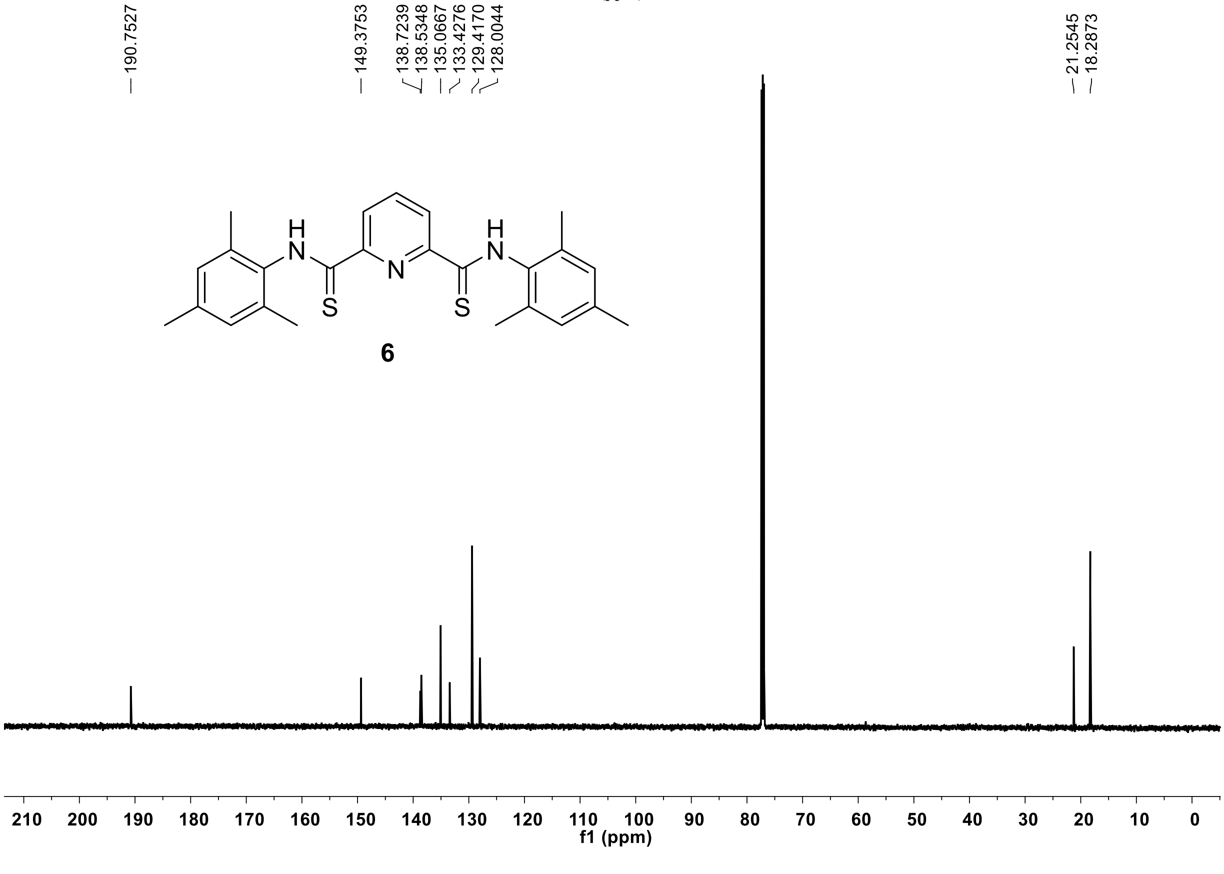

(4-Methoxyphenyl)phosphonothioic *O,O*-acid [9]

^{1}H NMR ($CDCl_3$, 400 MHz): δ = 7.83 (br, 2H), 6.95-6.81 (m, 2H), 4.55 (br, 2H), 3.81 (s, 3H).

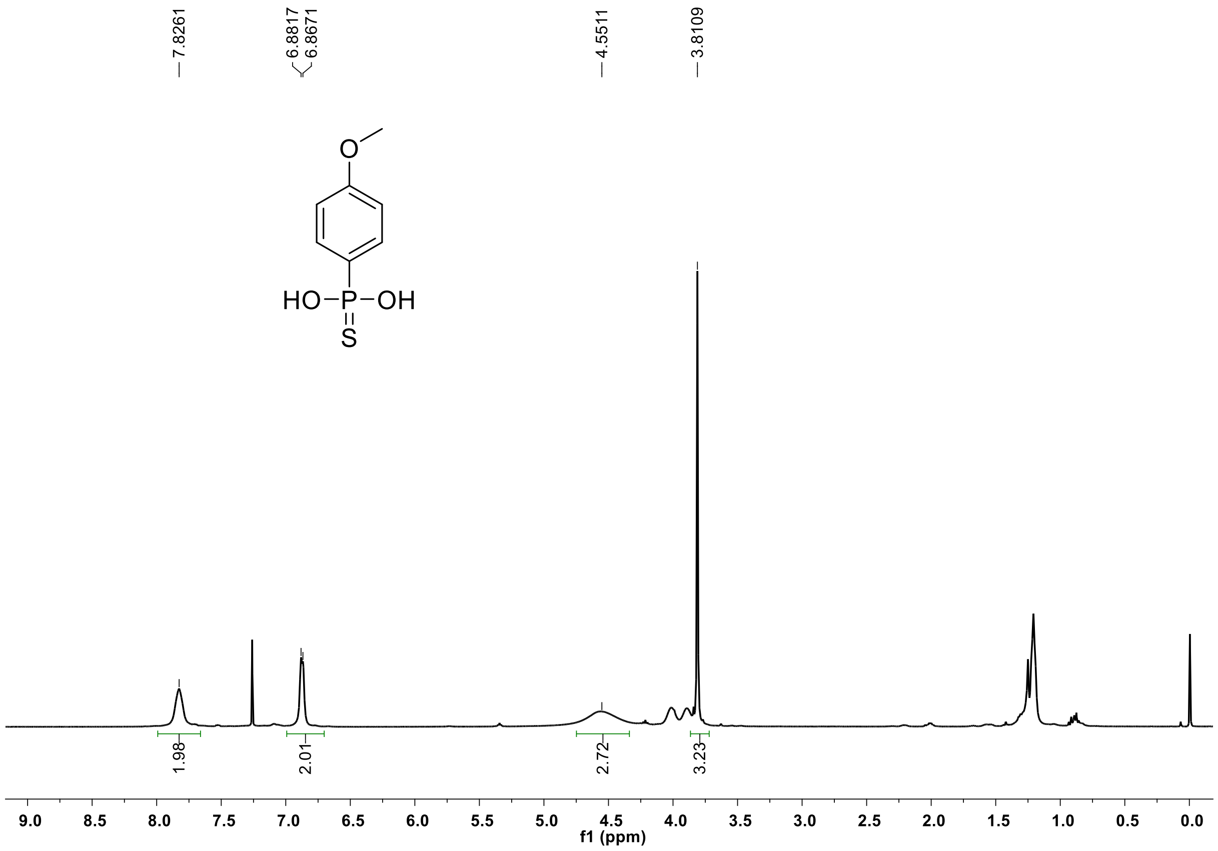

3. References

[1] N. T. Do, K. M. Tran, H. T. Phan, T. A. To, T. T. Nguyen, N. T. S. Phan, *Organic & biomolecular chemistry* **2019**, *17*, 8987-8991.

[2] P. Zhang, W. Chen, M. Liu, H. Wu, *The Journal of Organic Chemistry* **2018**, *83*, 14269-14276.

[3] M. Qiao, J. Zhang, L. Chen, F. Zhou, Y. Zhang, L. Zhou, Y. Wu, *Organic & biomolecular chemistry* **2019**, *17*, 3790-3796.

[4] B. V. Varun, A. Sood, K. R. Prabhu, *RSC Adv.* **2014**, *4*, 60798-60807.

[5] T. B. Nguyen, L. P. A. Nguyen, T. T. T. Nguyen, *Advanced Synthesis & Catalysis* **2019**, *361*, 1787-1791.

[6] H. K. Oh, S. K. Kim, H. W. Lee, I. Lee, *Journal of the Chemical Society, Perkin Transactions 2* **2001**, 1753-1757.

[7] C.-H. Yang, G.-J. Li, C.-J. Gong, Y.-M. Li, *Tetrahedron* **2015**, *71*, 637-642.

[8] J. Liu, H. Wang, H. Zhang, X. Wu, H. Zhang, Y. Deng, Z. Yang, A. Lei, *Chemistry - A European Journal* **2009**, *15*, 4437-4445.

[9] W. Przychodzeń, *European Journal of Organic Chemistry,* **2005**, 2005, 2002-2014
